# Do lower nitrogen fertilization levels require breeding of different types of cultivars in triticale?

**DOI:** 10.1007/s00122-021-04012-9

**Published:** 2021-12-27

**Authors:** Jan E. Neuweiler, Johannes Trini, Hans Peter Maurer, Tobias Würschum

**Affiliations:** 1 State Plant Breeding Institute, University of Hohenheim, 70599 Stuttgart, Germany; 2 Institute of Plant Breeding, Seed Science and Population Genetics, University of Hohenheim, 70599 Stuttgart, Germany

## Abstract

**Key message:**

The comparably low genotype-by-nitrogen level interaction suggests that selection in early generations can be done under high-input conditions followed by selection under different nitrogen levels to identify genotypes ideally suited for the target environment.

**Abstract:**

Breeding high-yielding, nitrogen-efficient crops is of utmost importance to achieve greater agricultural sustainability. The aim of this study was to evaluate nitrogen use efficiency (NUE) of triticale, investigate long-term genetic trends and the genetic architecture, and develop strategies for NUE improvement by breeding. For this, we evaluated 450 different triticale genotypes under four nitrogen fertilization levels in multi-environment field trials for grain yield, protein content, starch content and derived indices. Analysis of temporal trends revealed that modern cultivars are better in exploiting the available nitrogen. Genome-wide association mapping revealed a complex genetic architecture with many small-effect QTL and a high level of pleiotropy for NUE-related traits, in line with phenotypic correlations. Furthermore, the effect of some QTL was dependent on the nitrogen fertilization level. High correlations of each trait between N levels and the rather low genotype-by-N-level interaction variance showed that generally the same genotypes perform well over different N levels. Nevertheless, the best performing genotype was always a different one. Thus, selection in early generations can be done under high nitrogen fertilizer conditions as these provide a stronger differentiation, but the final selection in later generations should be conducted with a nitrogen fertilization as in the target environment.

**Supplementary Information:**

The online version contains supplementary material available at 10.1007/s00122-021-04012-9.

## Introduction

Nitrogen fertilization has become one of the main criticisms of modern agriculture in society. The application of high rates of nitrogen fertilizer in agriculture can have severe negative impacts on the environment and on ground and surface water, especially on aquatic ecosystems. The main reasons for this are nitrogen leaching caused by strong rainfalls, evaporation of gaseous nitrogen compounds from agricultural soils and canopies as well as high energy consumption for the production of mineral nitrogen fertilizers and as a result an increased emission of greenhouse gases (Ceotto 2005; David et al. 1997; Davidson 2009; Li et al. 2008). This has led to an increasingly restrictive legislation regulating nitrogen fertilization (Council Directive 91/676/EEC of 12 December (1991) concerning the protection of waters against pollution caused by nitrates from agricultural sources 1991, 2008; Düngegesetz 2009, 2020). On the other hand, there is the need to meet the demands of a growing world population despite reductions in arable land and increasingly unfavorable climatic conditions due to climate change.

The average proportion of applied nitrogen recovered is approximately 50% for the total dry matter harvested of small-grain cereals, and 33% for the harvested grain (Sylvester-Bradley and Kindred 2009; Raun and Johnson 1999). A possible solution to this is to improve the nitrogen use efficiency (NUE) of crops by breeding, in order to make better use of the applied fertilizer (Cormier et al. 2016). In general, NUE expresses the amount of grain harvested per unit nitrogen available to the plant. There are two main processes into which NUE can be partitioned: the nitrogen uptake efficiency, representing a genotype’s ability to acquire the available nitrogen from the soil into the plant and the nitrogen utilization efficiency, which quantifies the ability of a genotype to convert the absorbed nitrogen into grain yield (Moll et al. 1982). There are different morphological and physiological traits related to NUE, e.g., root morphology, delayed senescence and post-anthesis nitrogen uptake (Hirel et al. 2007; Spano et al. 2003; Triboi et al. 2006).

Cormier et al. (2014) tested 214 elite winter wheat cultivars under two nitrogen fertilization levels for 28 traits related to NUE. Genome-wide association mapping revealed a total of 333 QTL on several chromosomes, suggesting a complex genetic architecture. This was supported by findings of Guttieri et al. (2017), who evaluated 299 elite winter wheat cultivars and breeding lines under two levels of nitrogen fertilization for NUE and related traits to conduct genome-wide association mapping. This analysis revealed several QTL with small effect on chromosomes 1A, 1D, 2B, 2D and 4B, which is in line with findings of Cormier et al. (2014). Further studies on QTL detection of NUE and related traits in wheat support these results (Brasier et al. 2020; Monostori et al. 2017; Zhang et al. 2019).

In triticale, only a few studies on agronomic properties of nitrogen use efficiency have been published (Janušauskaitė 2013; Lewandowski and Schmidt 2006), as well as small-scale breeding experiments. Thiemt (2007) tested 36 triticale genotypes for nitrogen uptake and utilization efficiency and found significant genotypic variance for both traits, suggesting enough variance present for an improvement by breeding. A further study was conducted by Kutlu and Gülmezoğlu (2017) comprising 11 triticale genotypes and yielded similar results.

From a breeding point of view, the question arises how NUE and thus grain yield depend on the nitrogen fertilization level. Does the shift to lower nitrogen fertilization require different genetics and thus different genotypes to those selected for during the past decades? To the best of our knowledge, no large-scale experiment has yet been conducted to assess nitrogen use efficiency of triticale toward its improvement by breeding. In this study, we evaluated a diversity panel of 450 triticale genotypes under four nitrogen fertilizer levels in multi-environment field trials for grain yield (GY), protein content (PC) and starch content (SC), as well as for the derived traits protein yield (PY), nitrogen use efficiency (NUE) and nitrogen use efficiency for protein content (NUE_PC_). Our objectives were to (1) determine the variation, heritability and correlations between traits under different nitrogen fertilizer levels, as well as the interaction between genotype and nitrogen level, (2) evaluate the traits for long‐term genetic trends resulting from breeding progress, (3) uncover the genetic architecture of these traits and evaluate the potential of putative QTL for marker-assisted triticale breeding and (4) develop strategies for phenotypic selection of genotypes, suitable for specific nitrogen levels.

## Materials and methods

### Plant material and experimental design

In this study, a total of 450 diverse winter triticale (× *Triticosecale* Wittmack) genotypes of European origin were used, including registered cultivars ($n=126$) and advanced breeding material ($n=324$) representing the genetic diversity present in European triticale. All lines were provided by public institutions and private breeding companies.

For the field trials, 144 triticale genotypes were tested in 2018 under four different nitrogen fertilization levels at four locations. The set of genotypes was enlarged to 450 triticale genotypes and tested under the same nitrogen fertilization levels at the same four locations in 2019. In 2018, we used a partially replicated incomplete block design with 1.25 replications at two locations and a row–column design with 1.25 replications at the other two locations. In 2019, we used a row–column design with 1.2 replications at all four locations (Supplementary Table S1). Randomization and selection of the replicated lines was done using the software CycDesigN version 6.0 (Whitaker et al. 2002). Nitrogen fertilization treatments were applied in four blocks, in which all 144 genotypes in 2018 and all 450 genotypes in 2019 were included and replicated. This adds up to a total number of field plots per environment of 720 in 2018 and 2.160 in 2019 (Supplementary Fig. S1). Each field plot ranged in size from 5 to 10.5 m^2^, depending on the location. To account for the different plot sizes, grain yield was calculated as decitonnes per hectare [dt ha^−1^] with the formula: $\frac{GY plot [kg]}{plot size [{m}^{2}]}*100=GY [dt {ha}^{-1}]$. Nitrogen fertilization was calculated location-specific according to the latest fertilizer regulation (Verordnung zur Neuordnung der guten fachlichen Praxis beim Düngen 2017, 2020) by the German government. In brief, this regulation takes into account the average grain yield of triticale for the last three years at the respective environment, preceding crop, catch crop, organic fertilization in the last three years, humus content and the mineral nitrogen in the soil. The maximum legal amount of nitrogen which may be applied at each location was set as 100% level (N3) and represents the standard field practice of a conventional farmer. Further levels were set as a 70% level (N2) representing the amount of nitrogen applied by an organic farmer having a livestock of 1.5 cows per hectare farmland, a 40% level (N1) representing a low nitrogen environment and a 130% level (N4) representing a nitrogen oversupplied environment. Nitrogen fertilization was split in three applications, 50% of the total nitrogen demand at the beginning of the vegetation period, 25% at EC 31 (stem elongation, first node detectable on main stem) and 25% at EC 39 (flag leaf ligule just visible). The total amount of plant-available nitrogen at each environment can be seen in Supplementary Table S2. Tillage and crop protection were carried out at each experimental station according to the standard field practice. However, fungal plant diseases were strictly monitored and fungicide applications were made accordingly during the growing season to compensate for differences in resistance levels to fungal plant diseases among the triticale genotypes used. The environmental conditions can be seen in Supplementary Fig. S2.

To determine grain yield (GY in dt ha^−1^), the whole plots were harvested and grain yield was calculated at a moisture content of 14%. Protein content (PC in %) and starch content (SC in %) were measured by near infrared spectroscopy (NIRS) using a Polytec model PSS-X-212 with a wavelength spectrum from 1200 to 2400 nm running on software PSS-S-HOP (Polytec GmbH). Results of grain yield and protein content for the registered cultivars included in this study can be found in Supplementary Table S3.

### Calculation of indices

Protein yield (PY) was calculated on a plot basis as the product of GY and PC representing the amount of harvested protein in decitonnes per ha.

Nitrogen use efficiency (NUE) was calculated on a plot basis following the suggestion of Moll et al. (1982) as the quotient of GY and plant-available nitrogen representing the amount of grain produced in decitonnes per kilogram plant-available nitrogen.

Nitrogen use efficiency for protein content (nitrogen use efficiency_PC_; NUE_PC_) was calculated on plot basis as the quotient of PC and plant-available nitrogen representing the grain protein content produced in percent per kilogram plant-available nitrogen.

### Phenotypic data

The phenotypic data consisted of a row–column design at some environments and an incomplete block design at others, both with 1.2 or 1.25 replications, that were analyzed using a one-step procedure as suggested by Ogutu et al. (2011). For across-nitrogen level analysis, the following linear mixed model was applied:

1 $${y}_{ijklm/ijkm}=\mu +{g}_{i}+{e}_{j}+{N}_{k}+{(ge)}_{ij}+{(gN)}_{ik}+{(eN)}_{jk}+{(geN)}_{ijk}+{r}_{ljk}+{b}_{mjk}+{\varepsilon }_{ijklm/ijkm}$$

where ${{y}}_{{ijklm}/{ijkm}}$ was the phenotypic performance of the *i*th genotype at the *j*th environment within the *k*th nitrogen fertilizer level (with ${{y}}_{{ijklm}}$ corresponding to the row–column design and ${{y}}_{{ijkm}}$ to the incomplete block design), $\upmu$ the intercept, ${{g}}_{{i}}$ the effect of the *i*th genotype, ${{e}}_{{j}}$ the effect of the *j*th environment, ${{N}}_{{k}}$ the effect of the *k*th nitrogen fertilizer level, ${(\mathrm{ge})}_{{ij}}$ the genotype‐by‐environment interaction effect of the *i*th genotype and *j*th environment, ${(\mathrm{gN})}_{{ik}}$ the genotype‐by‐nitrogen fertilizer level interaction effect of the *i*th genotype and *k*th nitrogen fertilizer level, ${(\mathrm{eN})}_{{jk}}$ the environment‐by‐nitrogen fertilizer level interaction effect of the *j*th environment and *k*th nitrogen fertilizer level, ${(\mathrm{geN})}_{{ijk}}$ the genotype‐by‐environment-by-nitrogen fertilizer level interaction effect of the *i*th genotype, *j*th environment and *k*th nitrogen fertilizer level. For six out of eight environments, ${{r}}_{{ljk}}$ represents the *l*th row nested within the *j*th environment and *k*th nitrogen fertilizer level and ${{b}}_{{mjk}}$ is the *m*th column nested within the *j*th environment and *k*th nitrogen fertilizer level (the row–column design), whereas for two out of eight environments (the incomplete block design) ${{r}}_{{ljk}}$ is not defined and ${{b}}_{{mjk}}$ is the *m*th incomplete block nested within the *j*th environment and *k*th nitrogen fertilizer level (Supplementary Table S1). ${\upvarepsilon }_{{ijklm}/{ijkm}}$ was the residual associated with ${{y}}_{{ijklm}/{ijkm}}$.

For the within-nitrogen level analysis, the following linear mixed model was applied:

2 $${y}_{ijlm/ijm}={g}_{i}+{e}_{j}+{(ge)}_{ij}+{r}_{lj}+{b}_{mj}+{\varepsilon }_{ijlm/ijm}$$

where ${y}_{ijlm/ijm}$ was the phenotypic performance of the *i*th genotype at the *j*th environment (with ${{y}}_{{ijklm}}$ corresponding to the row–column design and ${{y}}_{{ijkm}}$ to the incomplete block design), $\mu$ the intercept, ${g}_{i}$ the effect of the *i*th genotype, ${e}_{j}$ the effect of the *j*th environment, ${(ge)}_{ij}$ the genotype‐by‐environment interaction effect of the *i*th genotype and *j*th environment. For six out of eight environments, ${r}_{lj}$ represents the *l*th row nested within the *j*th environment and ${b}_{mj}$ is the *m*th column nested within the *j*th environment, whereas for two out of eight environments (the incomplete block design) ${r}_{lj}$ is not defined and ${b}_{mj}$ is the *m*th incomplete block nested within the *j*th environment (Supplementary Table S1). ${\varepsilon }_{ijlm/ijm}$ was the residual associated with ${y}_{ijlm/ijm}$.

For across-nitrogen level analysis (Formula (1)), heterogeneous error variances were assumed for nitrogen fertilization levels within environments. For within-nitrogen level analysis (Formula (2)), heterogeneous error variances were assumed for single environments. Variance components were estimated with a full random model using restricted maximum likelihood (REML) and significance of variance components was determined by likelihood ratio tests (Stram and Lee 1994). Broad-sense heritability was estimated as suggested by Piepho and Möhring (2007) as:

3 $${h}^{2}=\frac{{\sigma }_{g}^{2}}{{\sigma }_{g}^{2}+ \overline{\nu }/2}$$

where ${\sigma }_{g}^{2}$ is the genotypic variance and $\overline{\nu }$ is the mean variance of a difference of two adjusted treatment means (BLUEs). For the calculation of the best linear unbiased estimators (BLUEs), the same models (1) and (2) were applied, but with genotype modeled as fixed effect. All statistical analyses were performed with the software package ASReml-R 3.0 (Gilmour et al. 2009) in the statistical software R 3.4.4 (R Core Team 2017).

### Genotypic data analyses

For 442 genotypes, genotypic data were available, generated by a genotyping-by-sequencing (GBS) approach (DArTseq) at Diversity Arrays Technology Pty. Ltd. (Canberra, Australia). Quality checks were performed, removing markers that showed more than 13% missing values or had a minor allele frequency smaller than 5%. After these quality checks, for 19,562 of the markers a map position was known on the A genome and for 22,110 markers on the B genome (Li et al. 2015). For the A and B genome, the wheat consensus map version 4.0 provided by Diversity Arrays Technology Pty. Ltd. (Canberra, Australia) was used, available at: https:/www.diversityarrays.com/technology-and-resources/genetic-maps/. In addition, a map position on the R genome was determined based on a segregating population using the MSTmap algorithm of the R package ASmap (Taylor and Butler 2017) resulting in 6641 markers after quality checks. For the R genome, the rye ‘Lo7’ assembly was used as reference (Rabanus-Wallace et al. 2021). Consequently, a map position was available for 48,313 unique markers which were used for genome-wide association mapping (33,992 dominant silico-DArTs and 14,321 SNPs). The CloneIDs of the silico DArT markers were given a ‘D’ and the SNP markers a ‘S’ prefix.

### Genome-wide association mapping

Genome-wide association mapping in the diversity panel was performed with the R package GenABEL (Aulchenko et al. 2007), using a linear mixed model that integrates a kinship matrix to correct for population stratification (Langer et al. 2014; Würschum and Kraft 2014; Yu et al. 2006). As significance threshold, we chose a Bonferroni-corrected significance level of *P* < 0.05. In the first step, the analysis was performed with all mapped and unmapped markers. To determine the most likely chromosomal positions of significantly associated unmapped markers, we evaluated their linkage disequilibrium (LD) with the mapped markers and placed them at the most probable position in a second step. For the calculation of the proportion of explained genotypic variance, marker data were imputed using Beagle 5.0 (Browning et al. 2018). After correction for collinearity by a joint fit in a linear model in the order of the strength of the association, significant QTL were labeled as ‘qChromosome’ followed by consecutive alphabetic letters, which were assigned to the QTL in the order of their proportion of explained genotypic variance, i.e., the QTL with the highest proportion of explained genotypic variance on chromosome 5A was designated q5A.A. All QTL were simultaneously fitted in a linear model in the order of the strength of the association to obtain the adjusted *R*^2^ values. The total proportion of the genotypic variance (${p}_{G}$) explained by all detected QTL was calculated from the ratio ${p}_{G}={R}_{adj}^{2}/{h}^{2}$, where ${h}^{2}$ refers to the heritability of the trait (Utz et al. 2000). Estimates of individual QTL were derived from the sums of squares of the QTL (${SS}_{QTL}$) in this linear model. In general, the α allele substitution effect can be expressed as:

4 $$\alpha =a\left[1+k\left({p}_{1}-{p}_{2}\right)\right]$$

where $a$ is the genotypic value of a locus, $k$ is the degree of dominance, and ${p}_{1}$ and ${p}_{2}$ are the allele frequencies. For an inbred line, the α allele substitution effect corresponds to the half of the value between the two genotypic classes of a QTL and was obtained by fitting the diagnostic marker of a QTL against the respective trait in a linear model; the resulting regression coefficient represents the α allele substitution effect (Lynch and Walsh 1998).

## Results

In this study, we analyzed a total of 450 triticale genotypes under four different nitrogen fertilization levels. The nitrogen levels were 40% (N1), 70% (N2), 100% (N3) and 130% (N4). We assessed grain yield, protein content and starch content in multi-environment field trials and calculated several indices to quantify the effect of different nitrogen fertilization on triticale. Considerable variation was found for all traits, as well as for the derived indices at all nitrogen fertilization levels. For all traits and nitrogen fertilization levels, the genotypic (${\sigma }_{g}^{2}$) and the genotype-by-environment interaction variance (${\sigma }_{g\times e}^{2}$) were highly significant (Table 1). The highest ratio between ${\sigma }_{g}^{2}$ and ${\sigma }_{g\times e}^{2}$ was found for grain yield with a ratio of 7.03 at nitrogen fertilization level N1 and the lowest ratio for protein yield with 1.93 at nitrogen fertilization level N2. For all traits as well as derived indices, the highest ratios were found in nitrogen fertilization level N1 except for protein yield, which had its highest ratio at nitrogen fertilization level N3. Moreover, the across-nitrogen level analysis revealed considerable and significant variation for the genotype-by-nitrogen fertilization interaction variance (${\sigma }_{g\times N}^{2}$), the environment-by-nitrogen fertilization interaction variance (${\sigma }_{e\times N}^{2}$) as well as the genotype-by-environment-by-nitrogen fertilization interaction variance (${\sigma }_{g\times e\times N}^{2}$). The ratio between ${\sigma }_{g}^{2}$ and ${\sigma }_{g\times N}^{2}$ ranged from 5.5 for protein yield to 47 for starch content. Estimates of heritability were moderate to high for all traits at all nitrogen fertilization levels and across them, ranging from 0.69 for protein yield at nitrogen fertilization level N1 to 0.95 for starch content in the across-nitrogen level analysis. The heritability estimates of each trait were similar between nitrogen fertilization levels.

**Table 1:** Summary statistics for the six investigated traits. Overall mean, range, genotypic (${\sigma }_{g}^{2}$), genotype-by-environment (${\sigma }_{g\times e}^{2}$), genotype-by-nitrogen (${\sigma }_{g\times N}^{2}$), genotype-by-environment-by-nitrogen (${\sigma }_{g\times e\times N}^{2}$) and error ($\overline{{\sigma }_{\epsilon}^{2}}$) variance components and heritabilities (${h}^{2}$) for six traits evaluated at 8 environments

	GY	PC	SC	PY	NUE	NUE_PC_
	[dt ha^−1^]	[%]	[%]	[dt ha^−1^]	[dt kg(N)^−1^]	[%(Protein) kg(N)^−1^]
Overall						
Min	51.55	10.76	65.10	7.89	0.44	0.07
Mean	100.01	11.74	69.67	11.82	0.65	0.08
Max	116.48	14.31	72.17	13.49	0.74	0.09
${\sigma }_{g }^{2}$	57.55***	0.22***	0.94***	0.33***	1.53 × 10^–3^***	6.28 × 10^–6^***
${\sigma }_{g\times e}^{2}$	18.12***	0.04***	0.15***	0.14***	5.50 × 10^–4^***	1.12 × 10^–6^***
${\sigma }_{g\times N}^{2}$	2.73***	0.01***	0.02***	0.06***	1.24 × 10^–4^***	9.28 × 10^–7^***
${\sigma }_{e\times N}^{2}$	42.71***	0.10***	0.16***	0.85***	7.80 × 10^–3^***	1.72 × 10^–5^***
${\sigma }_{g\times e\times N }^{2}$	2.30***	0.01***	0.03***	0.04***	4.05 × 10^–5^*	1.10 × 10^−7^ ns
${\overline{\sigma }}_{\epsilon}^{2}$	20.20	0.09	0.18	0.44	1.67 × 10^–3^	7.39 × 10^–6^
${h}^{2}$	0.91	0.93	0.95	0.83	0.89	0.90
N1						
Min	46.14	8.90	66.15	6.19	0.71	0.11
Mean	86.88	10.18	70.86	8.88	1.04	0.12
Max	103.58	13.17	73.11	10.12	1.20	0.15
${\sigma }_{g }^{2}$	36.57***	0.19***	0.93***	0.13***	3.87 × 10^–3^***	2.23 × 10^–5^***
${\sigma }_{g\times e}^{2}$	5.20***	0.02***	0.15***	0.05***	5.17 × 10^–4^***	2.92 × 10^–6^***
${\overline{\sigma }}_{\epsilon}^{2}$	29.84	0.12	0.18	0.57	4.54 × 10^–3^	1.69 × 10^–5^
${h}^{2}$	0.88	0.91	0.93	0.69	0.86	0.89
N2						
Min	43.09	10.02	65.15	6.02	0.43	0.07
Mean	99.49	11.20	70.06	11.11	0.67	0.08
Max	118.33	13.54	72.49	12.99	0.80	0.09
${\sigma }_{g }^{2}$	54.32***	0.20***	0.97***	0.27***	2.03 × 10^–3^***	9.37 × 10^–6^***
${\sigma }_{g\times e}^{2}$	17.19***	0.04***	0.17***	0.14***	9.28 × 10^–4^***	1.90 × 10^–6^***
${\overline{\sigma }}_{\epsilon}^{2}$	20.82	0.12	0.20	0.44	1.15 × 10^–3^	5.64 × 10^–6^
${h}^{2}$	0.86	0.89	0.93	0.71	0.82	0.89
N3						
Min	53.84	11.16	64.51	8.43	0.25	0.05
Mean	105.80	12.39	69.33	13.05	0.50	0.06
Max	124.03	14.78	72.13	15.13	0.58	0.07
${\sigma }_{g }^{2}$	66.38***	0.24***	1.00***	0.47***	1.39 × 10^–3^***	5.72 × 10^–6^***
${\sigma }_{g\times e}^{2}$	17.31***	0.07***	0.20***	0.17***	3.80 × 10^–4^***	1.57 × 10^–6^***
${\overline{\sigma }}_{\epsilon}^{2}$	26.69	0.08	0.19	0.52	8.03 × 10^–4^	2.11 × 10^–6^
${h}^{2}$	0.89	0.88	0.92	0.80	0.88	0.88
N4						
Min	55.65	12.13	64.31	9.47	0.20	0.04
Mean	107.95	13.21	68.42	14.21	0.39	0.05
Max	130.26	15.30	70.91	17.04	0.47	0.06
${\sigma }_{g }^{2}$	71.14***	0.23***	0.89***	0.69***	9.27 × 10^–4^***	3.22 × 10^–6^***
${\sigma }_{g\times e}^{2}$	24.94***	0.06***	0.21***	0.28***	3.64 × 10^–4^***	8.11 × 10^–7^***
${\overline{\sigma }}_{\epsilon}^{2}$	24.06	0.08	0.16	0.47	4.30 × 10^–4^	1.16 × 10^–6^
${h}^{2}$	0.86	0.89	0.91	0.81	0.85	0.89

Traits are GY, grain yield; PC, protein content; SC, starch content; PY, protein yield; NUE, nitrogen use efficiency; NUE_PC_, nitrogen use efficiency for protein content

***Significant at the 0.001 probability level

*Significant at the 0.05 probability level

**Fig. 1:**
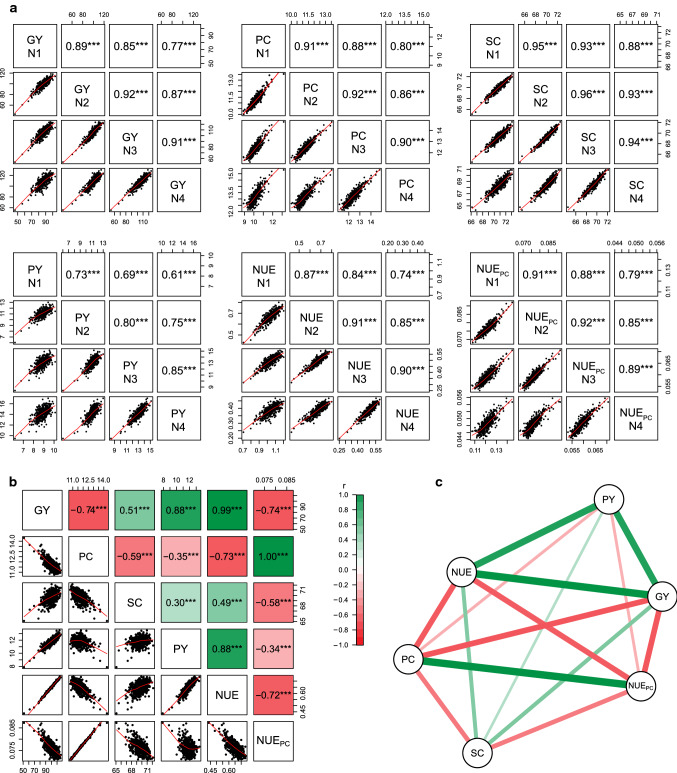
Correlations among nitrogen levels and traits. **a** Phenotypic correlations and scatterplots between nitrogen fertilization levels within traits. **b** Phenotypic correlations and scatterplots of the trait BLUEs across-nitrogen levels. **c** Network analysis of the same BLUEs. Traits are GY, grain yield; PC, protein content; SC, starch content; PY, protein yield; NUE, nitrogen use efficiency; NUE_PC_, nitrogen use efficiency for protein content

The analysis of correlations between the four nitrogen fertilization levels revealed significant positive correlations between all nitrogen fertilization levels for all traits (Fig. 1a). The smaller the difference between the nitrogen fertilization levels, the higher the correlation. For example, nitrogen fertilization levels N2 and N3 showed a correlation of *r* = 0.92*** for grain yield, whereas the correlation between nitrogen fertilization levels N1 and N4 was only *r* = 0.77***. The same trend was observed for all other traits. The across-nitrogen level analysis revealed significant coefficients of correlation for all trait combinations (Fig. 1b, c). Grain yield was negatively correlated with protein content (*r* = -0.74***), but positively correlated with starch content (*r* = 0.51***), whereas protein content and starch content were negatively correlated (*r* = -0.59***). The derived indices protein yield and nitrogen use efficiency were positively correlated with grain yield (*r* = 0.88**, *r* = 0.99***, respectively), whereas the derived index nitrogen use efficiency for protein content showed a negative correlation with grain yield (*r* = -0.74***). The opposite picture was observed for protein content and its correlation with the derived indices protein yield, nitrogen use efficiency and nitrogen use efficiency for protein content (*r* = -0.35***, *r* = -0.73***, *r* = 1.00***, respectively). The indices protein yield and nitrogen use efficiency showed a highly positive correlation with each other (*r* = 0.88***), whereas both were significantly negative correlated with nitrogen use efficiency for protein content (*r* = -0.34***, *r* = -0.72***, respectively). The analysis within single nitrogen levels yielded similar results (data not shown).

**Fig. 2:**
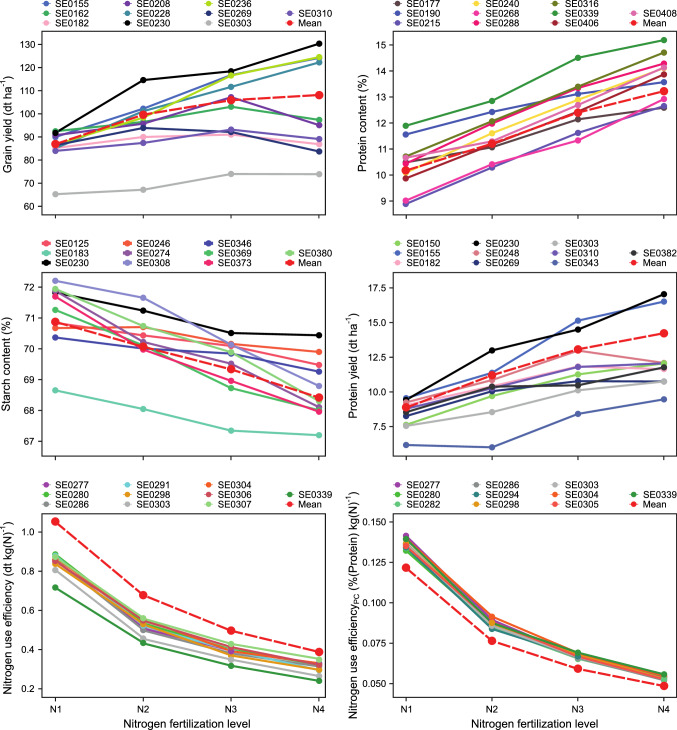
Performance of the ten genotypes having the highest genotype-by-nitrogen level interaction variance (${\sigma }_{g\times N}^{2}$) for the respective traits over the four different nitrogen fertilization levels. The dashed red line indicates the mean performance of all genotypes included in this study

To determine the reaction of the individual genotypes to varying nitrogen availability, we fitted the genotype-by-nitrogen fertilization interaction variance for every genotype. For every trait, the ten genotypes showing the highest genotype-by-nitrogen fertilization interaction variance were plotted for graphical analysis (Fig. 2). This analysis revealed considerable differences between genotypes’ reaction to varying nitrogen availability and between traits. The ten most extreme genotypes for grain yield were either very high or very low yielding ones. For nitrogen use efficiency, all ten genotypes showed a performance below average, whereas for nitrogen use efficiency for protein content all ten genotypes showed a performance above average.

**Fig. 3:**
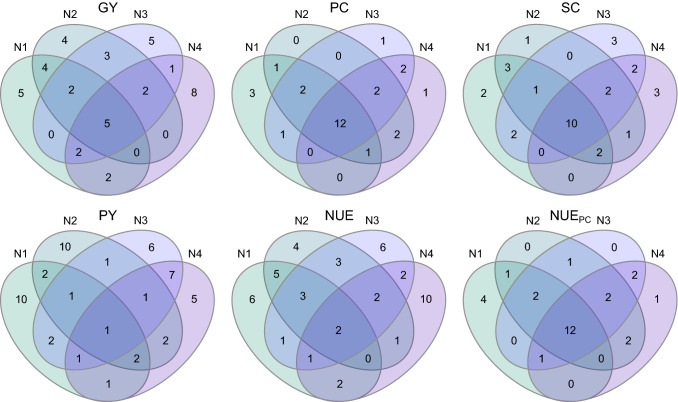
Venn diagrams of the twenty best performing genotypes for the respective traits over the four nitrogen fertilization levels. Traits are: GY, grain yield; PC, protein content; SC, starch content; PY, protein yield; NUE, nitrogen use efficiency; NUE_PC_, nitrogen use efficiency for protein content

To further analyze the performance-stability of genotypes under varying nitrogen availability for a specific trait, we compared the 20 best performing genotypes of every nitrogen fertilization level (Fig. 3). For the traits protein content, starch content and nitrogen use efficiency for protein content 12, 10 and 12 genotypes were in common between nitrogen fertilization levels, respectively. In contrast, grain yield, protein yield and nitrogen use efficiency only had 5, 1 and 2 genotypes in common between nitrogen fertilization levels, respectively.

**Fig. 4:**
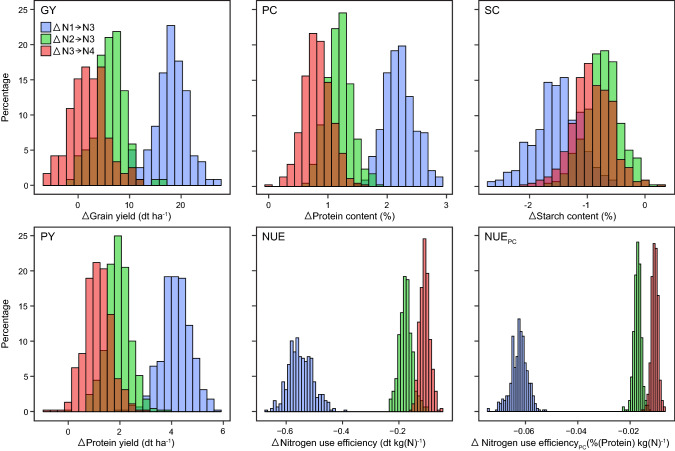
Trait differences among nitrogen levels. Histograms of the effect of additional fertilizer on the performance of 450 triticale genotypes. The legal maximum for conventional farmers in Germany (nitrogen fertilization level N3) is used as a reference

To assess the effect of nitrogen application in comparison with the nitrogen fertilization level N3, we calculated the difference in performance between nitrogen fertilization level N1 and N3 (ΔN1→N3), N2 and N3 (ΔN2→N3) and N3 and N4 (ΔN3→N4) (Fig. 4). The analysis showed that all genotypes increased their trait values for protein content and protein yield with increased nitrogen fertilization. The opposite was observed for starch content, nitrogen use efficiency and nitrogen use efficiency for protein content. Grain yield showed an increase in performance for ΔN1→N3 and ΔN2→N3 for all genotypes, whereas for ΔN3→N4, only a fraction of genotypes increased their grain yield.

We further analyzed the relative effect of nitrogen application in comparison with the nitrogen fertilization level N3 on registered cultivars released during the last 20 years (Supplementary Fig. S3). For grain yield, protein content, protein yield and nitrogen use efficiency, the analysis revealed a slight increase of ΔN over the last 20 years. The only significant changes in this analysis showed protein content for ΔN3→N4 and protein yield for ΔN2→N3. On the other hand, starch content showed a slight decrease for ΔN1→N3 and ΔN3→N4, whereas ΔN2→N3 showed a slight increase over the last 20 years; however, these changes were not significant. For nitrogen use efficiency for protein content, there was no considerable change over the last 20 years.

**Fig. 5:**
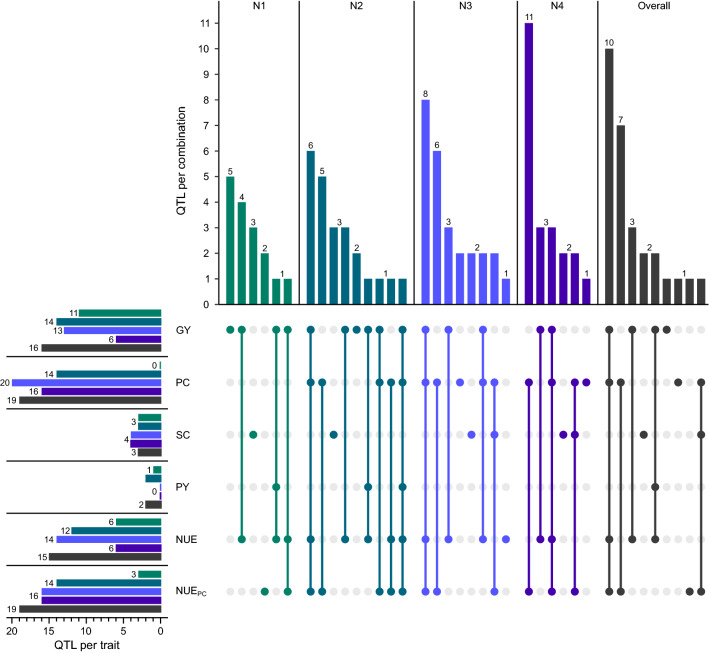
Number of QTL per trait and trait combinations of pleiotropic QTL within and across the different nitrogen fertilization levels. Traits are: GY, grain yield; PC, protein content; SC, starch content; PY, protein yield; NUE, nitrogen use efficiency; NUE_PC_, nitrogen use efficiency for protein content

**Fig. 6:**
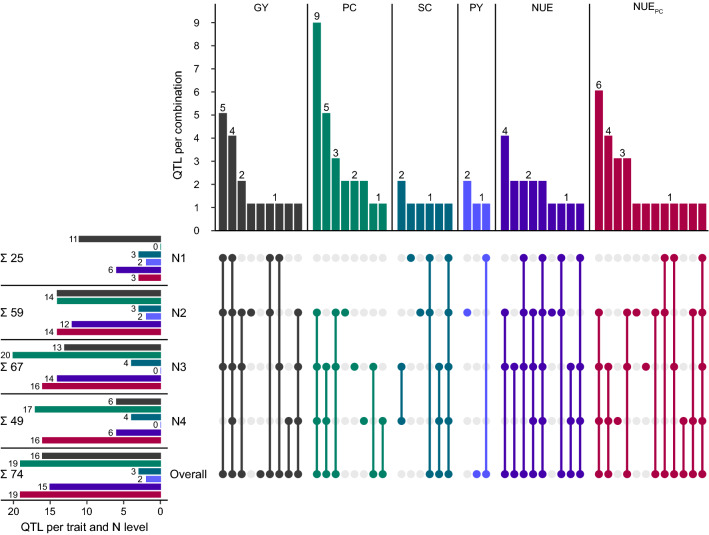
Number of QTL per nitrogen fertilization level and combination of nitrogen fertilization levels in which QTL are identified for the respective traits. Traits are: GY, grain yield; PC, protein content; SC, starch content; PY, protein yield; NUE, nitrogen use efficiency; NUE_PC_, nitrogen use efficiency for protein content

To uncover the effect of varying nitrogen availability on the genetics underling the analyzed traits, we performed genome-wide association mapping within all four nitrogen fertilization levels as well as across nitrogen fertilization levels. This identified QTL for all six traits within all nitrogen fertilization levels, except for protein content in nitrogen fertilization level N1 and protein yield in nitrogen fertilization level N3 and N4 (Table 2, Figs. 5, 6, Supplementary Table S2). Most of the putative QTL explained only a small to medium proportion of the genotypic variance (${p}_{G}$) of the respective trait. The total proportion of genotypic variance explained by the identified QTL ranged from 10.23% for protein yield in nitrogen fertilization level N1 to 59.57% for grain yield in nitrogen fertilization level N2. On the other hand, across all analyses within and across N levels, nine putative QTL explained more than 10% of the genotypic variance for at least one trait. Moreover, three of those explained more than 20% of the genotypic variance for at least one trait and can be classified as medium-effect QTL. Many of the QTL were in common between traits, as 30 out of 36 detected unique QTL showed pleiotropy for at least two traits (Fig. 5). This analysis further showed that the highest number of QTL was found within moderate nitrogen fertilization levels N2 and N3 for all traits. The across-nitrogen level analysis yielded similar results to nitrogen fertilization levels N2 and N3 or an even higher number of QTL. Moreover, many QTL were identified in several nitrogen fertilization levels and only a few QTL appear to be specific to one nitrogen fertilization level (Fig. 6).

**Table 2:** Results of genome-wide association mapping. Number of QTL, chromosomes with QTL and proportion of total genotypic variance explained (${p}_{G}$) for the respective trait across and cross nitrogen level analysis. Chromosomes with QTL ≥ 20% ${p}_{G}$ are underlined

	No. of QTL			Chromosomes with QTL	${p}_{G}$ total
	${p}_{G}$		All		
	10% − 20%	≥ 20%			
N1					
Grain yield	3	0	11	1B 4A 5A 5B 6B 6R 7B	45.13
Starch content	0	0	3	4A 5B	12.07
Protein yield	1	0	1	1B	10.23
Nitrogen use efficiency	2	0	6	1B 4A 5A 6R 7B	42.45
Nitrogen use efficiency_PC_	1	0	3	1B 2A 2B	20.81
N2					
Grain yield	1	1	14	1B 2B 4B 5A 5B 6B 6R 7A 7B	59.57
Protein content	0	1	14	1A 1B 2B 3A 4A 4B 5A 5B 6B	39.01
Starch content	0	0	3	1A 4A 5B	18.74
Protein yield	1	0	2	5A 5B	18.53
Nitrogen use efficiency	0	1	12	1B 4A 4B 5A 5B 6B 6R 7A 7B	51.33
Nitrogen use efficiency_PC_	0	1	14	1A 1B 2B 3A 4A 4B 5A 5B 6B	38.43
N3					
Grain yield	1	1	13	1B 2B 4A 4B 5A 5B 6B 6R	50.95
Protein content	0	1	20	1A 1B 2A 2B 3A 4A 4B 5A 5B 6B	47.92
Starch content	1	0	4	3A 5A 5B	17.21
Nitrogen use efficiency	3	0	14	1B 2B 4A 4B 5A 5B 6B 6R	51.83
Nitrogen use efficiency_PC_	0	1	16	1A 1B 2A 3A 4A 4B 5A 5B	45.79
N4					
Grain yield	1	1	6	1B 4A 5B 6R	38.71
Protein content	1	0	17	1A 1B 3A 4A 4B 5A 5B 5R 6B	53.32
Starch content	0	0	4	3A 5A 5B	18.44
Nitrogen use efficiency	0	1	6	1B 4A 5B 6R	39.29
Nitrogen use efficiency_PC_	1	0	16	1A 1B 3A 4A 4B 5A 5B 5R 6B	52.10
Overall					
Grain yield	3	0	16	1B 2B 4A 4B 5A 5B 6B 6R 7B	53.04
Protein content	0	1	19	1A 1B 2A 2B 3A 4A 5A 5B 6B	45.16
Starch content	0	0	3	4A 5A 5B	13.28
Protein yield	1	0	2	1B 6R	17.63
Nitrogen use efficiency	0	1	15	1B 2B 4A 4B 5A 5B 6B 6R 7B	55.34
Nitrogen use efficiency_PC_	0	1	19	1A 1B 2A 2B 3A 4A 4B 5A 5B 6B	46.45

## Discussion

It is well known that excessive nitrogen fertilization has severe impacts on ecosystems and the environment. Consequently, on the one hand, a responsible use of nitrogen fertilizers in agriculture is necessary, and on the other hand, we have to maintain the yields at the highest possible level in order to meet the demands of a growing world population. To resolve this contradiction, we need to focus on breeding resource-efficient cultivars, which make better use of the available nitrogen. To this end, we evaluated a diversity panel of 450 triticale genotypes, consisting of registered cultivars and advanced breeding lines of the past 20 years, under four nitrogen fertilization levels in multi-environment field trials in 2018 and 2019 for grain yield, protein content and starch content, as well as NUE and further indices to assess the possibility to improve nitrogen use efficiency of triticale by breeding. Furthermore, we performed genome-wide association mapping to uncover the genetic architecture underlying these traits and to evaluate the potential of marker-assisted breeding for their improvement. With this study, we aim to promote the breeding of new and resource-efficient cultivars of triticale and other small-grain cereals and offer breeders a breeding scheme that can be integrated into existing programs and thus also has a practical benefit for a modern and sustainable agriculture.

### Phenotypic variation and selection strategies—all for one, one for all?

The general trend shown by the genotypes included in our study was that the higher the amount of nitrogen available to the plants, the higher the grain yield (Table 1, Fig. 2). This is as expected, but to achieve today’s goals in agriculture, we are in the need to minimize our fertilizer input while maintaining grain yields on a stable level or even increase them. Thus, the goal is to identify genotypes that behave differently and do not follow the observed general trend. At the phenotypic level, there are several options to do so. The most common way among breeders is to simply select the best performing genotype for a given trait, usually focusing on grain yield. This is a valid approach if selecting in the target environment, but maybe less so when the target environment or conditions differ from those used for selection. This is shown by the correlations between the different nitrogen fertilization levels, which become lower the more different the nitrogen fertilization levels are. Consequently, it becomes less likely to identify the best performing genotype under lower N levels when selecting under high N conditions (Fig. 1). This is also evident when looking at the top 20 genotypes of each nitrogen fertilization level (Fig. 3). Most genotypes that performed well in one nitrogen level also performed well in the others, as reflected by the small values of the genotype-by-nitrogen interaction variance (${\sigma }_{g\times N}^{2}$) (Table 1). Thus, it is possible to select well performing genotypes for another target nitrogen environment by selecting only in one nitrogen fertilization level. However, to select the best ones, it is necessary to select under conditions specific to the target environment. This is in accordance with findings of Sattelmacher et al. (1994) who concluded that the direct selection on grain yield within the target nitrogen environment is the best solution to identify the best performing genotypes.

Another approach is to identify genotypes that deviate from normal behavior. For this, we estimated the genotype-by-nitrogen interaction variance of each genotype and selected those having the highest values (Fig. 2). Besides genotypes which perform very well or very poor at certain nitrogen fertilization levels, this approach mainly identified stable genotypes. In case of grain yield, stable genotypes mostly performed poorly and are for this reason not of further interest to breeders, but especially for the quality-related traits protein content and starch content we identified stable genotypes on an above-average performance level. Such stable, as well as top performing genotypes could serve as parental components for future programs to breed nitrogen-efficient cultivars.

Further, we assessed the reaction of the genotypes to additional nitrogen fertilizer by evaluating the differences (Δ) between the nitrogen fertilization level N3, representing the standard fertilization level of a conventional farmer in Germany, and the other nitrogen fertilization levels N1, N2 and N4 (Fig. 4). This clearly showed the difference between genotypes’ reaction to additional fertilizer availability. Some genotypes have an above-average ability to transform the additional nitrogen into grain yield. The same strong effect is evident for protein content and the index protein yield, whereas starch content showed only a small reaction. This can be explained by the chemical composition of starch, as it does not contain nitrogen and its formation is therefore less dependent of the availability of nitrogen. Interestingly, about half of the genotypes were not able to transform the additional nitrogen from nitrogen fertilization level N3 to N4 into grain yield, indicating that the site-specific legal nitrogen fertilization limit, represented by nitrogen fertilization level N3, was well chosen by authorities (Verordnung zur Neuordnung der guten fachlichen Praxis beim Düngen 2017, 2020).

For most of the traits, there is only a slight, mostly nonsignificant temporal trend in the exploitation of additional nitrogen fertilizer recognizable over the past 20 years (Supplementary Fig. S3). The only significant changes are present for protein content ΔN3→N4 and protein yield ΔN2→N3. However, for most traits and deltas an increase is recognizable, suggesting modern cultivars to be superior and have higher nitrogen use efficiency. This is in line with findings of Voss-Fels et al. (2019) who tested a large set of wheat cultivars, registered over the last 50 years, for their performance under low- and high-input scenarios, concluding modern cultivars to be superior in both scenarios. Even if our results are less pronounced, probably due to the shorter period of breeding progress represented by the cultivars, the temporal trend detected in our study is in line with the results of Voss-Fels et al. (2019). The inclusion of older material might have resulted in a higher variation in this study, but it must be noted that triticale has a much shorter history of intensive breeding than wheat and the possible benefits derived from old triticale material would have been negligible.

Our results suggest that the ongoing selection for higher grain yield is the major driver to improve cultivars’ nitrogen use efficiency, as there is the indirect selection of genotypes exploiting the available nitrogen in the most efficient way, which is in line with findings in wheat and barley (Sylvester-Bradley and Kindred 2009; Voss-Fels et al. 2019). To increase quality parameters, such as protein content or starch content, it is not recommended to select simply the best performing genotype, as those traits are either low correlated to grain yield in case of starch content, or even negatively correlated in case of protein content (Fig. 1). A more efficient way seems to be the use of indices accounting for both traits for a more balanced selection (Neuweiler et al. 2021; Rapp et al. 2018). In case of triticale, the increase in nitrogen use efficiency for protein content is less important as commodity markets do not appreciate high protein contents in triticale grain. Moreover, it is currently cheaper for farmers to compensate low protein levels in triticale grain by protein sources such as soybean than to grow high-protein triticale cultivars (Neuweiler et al. 2021). However, the situation is about to change as several European countries already included or will include protein content as a factor in the registration process of new triticale cultivars. To be prepared for possible new market situations and applications for high-protein triticale cultivars, a base selection for increased nitrogen use efficiency for protein content could pay off for the breeder. In conclusion, phenotypic selection methods have enough potential to identify genotypes suitable to improve the nitrogen use efficiency of triticale by breeding. Moreover, our results show that the triticale breeding pool harbors enough variation on a high level of heritability for all traits, providing the opportunity for further improvement (Table 1).

### Genetic architecture—QTL effects in two dimensions

We performed genome-wide association mapping within the single nitrogen fertilization levels, as well as across nitrogen levels. This analysis revealed a complex genetic architecture with many small-effect QTL and a high level of pleiotropy for all investigated traits. Only nine out of 36 identified unique QTL explained more than 10% of the genotypic variance, whereas three of them explained more than 20% of the genotypic variance and can be classified as medium-effect QTL (Table 2, Supplementary Table S4). Those are q1B.B, a pleiotropic QTL affecting protein content and NUE_PC_ located at the start of chromosome 1B, q5B.B a pleiotropic QTL affecting grain yield and NUE, as well as q5B.C, also a pleiotropic QTL affecting grain yield, protein yield and NUE. Both, q5B.B and q5B.C are located at the end of chromosome 5B. These findings are in accordance with previous results in triticale (Neuweiler et al. 2020, 2021) and with findings of Kuchel et al. (2007) and Quarrie et al. (2005) in wheat, who identified grain yield QTL on chromosome 5B. Monostori et al. (2017) detected NUE-related QTL in the same region of chromosome 5B in wheat. Further, Cormier et al. (2014) suspected an important locus on chromosome 5B that is related to NUE in wheat. A potential candidate gene for this QTL could be *UDP-GP* a gene coding for UDP-glucose phosphorylase involved in starch synthesis (Quraishi et al. 2011). Jiang et al. (2004) showed this gene to be affected by nitrogen supply and remobilization, and therefore, this gene could be directly connected to NUE.

Indicated by the high significant correlations between most traits, the level of pleiotropy was very high. Of the 36 unique putative QTL, 30 showed pleiotropy for at least two traits (Fig. 5), which can have different reasons. The high number of pleiotropic QTL between grain yield and protein content is due to the negative relationship between the two traits, as discussed in detail for triticale (Neuweiler et al. 2021) and wheat (Rapp et al. 2018; Thorwarth et al. 2018, 2019). The high number of pleiotropic QTL between grain yield and derived NUE, as well as protein content and derived NUE_PC_ can partially be explained by the mathematical derivation of the two indices. Further, the physiological basis of grain yield and protein formation is closely linked to the plants’ ability to exploit the available nutrients. The same pattern of colocalized QTL for protein content and NUE_PC_ was found by Cormier et al. (2014) and the close relation between grain yield and NUE was suggested by numerous studies (Brasier et al. 2020; Hitz et al. 2017).

There is not only a joint identification of QTL between traits, but also between QTL for different nitrogen fertilization levels (Fig. 6). Most of the putative QTL are present over a wide range of nitrogen fertilization levels, only 13 QTL were detected in only one N level. These results suggest a stable physiological mechanism in the formation of grain yield and grain composition. One example for such a stable QTL is q6R.A explaining 11.28%, 13.26% 12.09% and 10.32% of the genotypic variance of grain yield within nitrogen fertilization levels N1 to N4, respectively. In contrast, q5B.B was not significant within nitrogen fertilization level N1 and explained 4.66%, 23.05% and 20.52% of the genotypic variance of grain yield within nitrogen fertilization levels N2 to N4, respectively. Such stable QTL as q6R.A are potential candidates for marker-assisted selection to improve NUE in general, whereas QTL as q5B.B serve only as potential candidates for specific nitrogen environments. However, the total amount of genotypic variance explained jointly by all putative QTL for the single traits was of small to medium magnitude and ranged between 10.23% for protein yield in nitrogen fertilization level N1 to 59.57% for grain yield in nitrogen fertilization level N2. The complex genetic architecture of these traits with the small number of detected medium-effect QTL, together with the plasticity of explained variance of many QTL between N levels, the high level of pleiotropy and the high frequency of the positive allele for most of the putative QTL and therefore the high fixation of these QTL in the population, limits the potential of marker-assisted selection. A possible alternative could be the use of genomic selection. However, especially the plasticity of explained variance between N levels for specific QTL must be taken into account also for this approach. When setting up field trials for genomic selection, the environmental conditions for the training set and the prediction set should be identical in order to eliminate the observed plasticity of QTL effects between N levels to obtain reliable predictions. Thus, training sets specific for the targeted N fertilization level would be required, which warrants further research.

### Implications for breeding

Breeding for nitrogen-efficient cultivars is not trivial, as traits known to be related to NUE are difficult or expensive to phenotype as they are mostly related to root morphology or physiological pathways. Therefore, a careful preselection of the parental components is crucial for the success of the breeding program. Choosing the right parents to start a breeding cycle, for example, having the desired root morphology, will increase the probability to select nitrogen-efficient genotypes in their progeny without the need to phenotype root morphology in the offspring, as root morphology is only a tool to increase NUE, not the target trait.

Another important aspect is the environment to select in. In practice, most breeding companies select within high-input environments, comparable to conventional agriculture. Cultivars resulting from such a program are performing superior in high-input environments, but now with changing policies and legislation should also show a superior performance in medium- or low-input environments. Our results show that this is true in general, as most of the genotypes performing well in one nitrogen level also performed well in another nitrogen level. This was also indicated by the high correlations between nitrogen fertilization levels within traits and the comparably low genotype-by-nitrogen level interaction variance. However, the best performing genotype was always different and would be missed if selecting under only one N level. A selection strategy resulting from this is to carry out early generation field trials under conventional, high-input conditions with moderate selection intensity followed by a selection within different nitrogen levels in later generations to select the best performing genotypes for the target market. An argument for first selecting under high N conditions are the genotypic variances. For grain yield, for example, the genotypic variance doubled from the lowest nitrogen fertilization level N1 to the highest level N4, which makes it more likely to identify superior genotypes in early generations within high-input environments. This becomes obvious when looking at the response to selection. In our experiment, the response to selection (${R}_{s}$) adds up to 9.92 dt ha^−1^ selecting the 10% best genotypes for grain yield within-nitrogen fertilization level N1, whereas selecting the 10% best genotypes for N1 within standard nitrogen fertilization level N3, the response to indirect selection is 11.43 dt ha^−1^.

Another component in the breeding of N-efficient cultivars could be the use of hybrid cultivars. Prey et al. (2019) compared four hybrid and nine line winter wheat cultivars for nitrogen-efficiency related traits, showing that hybrid cultivars are superior in converting the available nitrogen into grain yield. However, the benefits of hybrid cultivars are countered by the general disadvantages of hybrid cultivars in small-grain cereals of high seed costs and low commercial heterosis.

A way to indirectly increase the NUE of small-grain cereals is resistance breeding. Only healthy plants can make full use of the available nitrogen and assure the translocation of starch and protein into the developing grains. In the present study, we eliminated this factor by a strict fungicide management, but with regard to organic agriculture it is of great importance to breed disease resistant cultivars. This will be even more important in the future, as not only the environmental problems associated with fertilization are controversial in society, but also the use of pesticides.

The use of molecular markers is questionable as there are no large-effect QTL known to be associated with NUE. Further, our results show that the effect of putative QTL also depends on the nitrogen fertilization level, making it even more difficult to identify reliable candidates for marker-assisted selection, which if used at all, would have to be N-level specific. We therefore suggest precise phenotyping and careful selection of parental components followed by phenotypic multistage selection within high-input environments in early generations and different nitrogen-environments in later generations to identify superior genotypes within the target environment as the optimum method for breeding of nitrogen use-efficient cultivars. Potentially, this breeding scheme might be assisted by genomic approaches, particularly genomic selection, to predict the performance of the early generation candidates in nitrogen-environments where they are not tested. This could increase the probability to advance the most promising ones to the later generations where they are tested in their target environment or even allow the selection of lines that perform well over a larger range of N levels, but this requires further research.

### Ecological considerations

In the temperate zone, the greatest negative impact of excessive nitrogen fertilization comes from nitrogen leaching. In our study, we mostly found low amounts of post-harvest soil mineral nitrogen in all locations and both years of about 30 kg N ha^−1^ for nitrogen fertilization level N3, representing the maximum legal amount of nitrogen which may be applied at each location (Supplementary Table S2). Only location Moosburg showed higher levels of about 60 kg N ha^−1^, which can be explained by the exceptionally dry conditions during the experiments (Supplementary Fig. S2). In general, soil mineral nitrogen amounts lower than 26–38 kg N ha^−1^, depending on the soil type, are not considered to be prone to leaching, concluding triticale to be a sustainable crop with regard to its nitrogen balance (Gaines and Gaines 1994; Center for Agricultural Technology Augustenberg 2014). Besides grain yield, it has been shown that there is no economic benefit of excessive nitrogen fertilization to increase the grain protein content of triticale as animal feed (Neuweiler et al. 2021). Taking into account the high nitrogen recovery of triticale, the low grain yield increase between nitrogen fertilization levels N2 and N3 (Table 1), and the fact that an increased nitrogen fertilization in favor of a higher grain protein content is of no economic benefit, reduced nitrogen applications comparable to nitrogen fertilization level N2 are sufficient for an economical and ecological cultivation of triticale.

## Conclusions

In order to reduce the negative impact of excessive nitrogen fertilization on the environment and to ensure a sufficient food production to feed a growing world population, it is crucial to improve the nitrogen use efficiency of modern cultivars. The genetic architecture of nitrogen use efficiency and related traits is characterized by many small-effect QTL and a high level of pleiotropy. Further, the effect of the putative QTL is dependent on the level of nitrogen fertilization, which additionally hampers the use of marker-assisted selection for nitrogen use efficiency and related traits in triticale breeding. Besides marker-assisted breeding, the genotypic variation found in our study was significant and of sufficient magnitude to be able to further increase the performance of new triticale cultivars by phenotypic selection. The analysis of temporal trends showed that new cultivars are superior in their ability to exploit the available nitrogen which suggests a continuous improvement of nitrogen use efficiency by selecting for higher grain yields. Our results also showed that the best performing genotypes within the single nitrogen fertilization levels were always different. In conclusion, we suggest that the direct phenotypic selection on grain yield within the target environment is the method of choice to identify superior genotypes and to increase the nitrogen use efficiency of triticale.

## Supplementary Information

Below is the link to the electronic supplementary material.

Supplementary file1 (PDF 1099 kb)

Supplementary file2 (XLSX 40 kb)

## Data Availability

The datasets generated during and analyzed during the current study are available from the corresponding author on reasonable request.
