# Supplementary material for: Do lower nitrogen fertilization levels require breeding of different types of cultivars in triticale?
Source: Theor Appl Genet. 2021 Dec 27;135(3):993–1009. doi: 10.1007/s00122-021-04012-9 (PMC8942957; doi:10.1007/s00122-021-04012-9)
Supplement: Supplementary file 1 — Supplementary file1 (PDF 1099 kb) [file 122_2021_4012_MOESM1_ESM.pdf]

Do lower nitrogen fertilization levels require breeding of different types of cultivars in triticale?

Jan E. Neuweiler[1], Johannes Trini[1], Hans Peter Maurer[1*], Tobias Würschum[2]

[1]State Plant Breeding Institute, University of Hohenheim, 70599 Stuttgart, Germany
[2]Institute of Plant Breeding, Seed Science and Population Genetics, University of Hohenheim, 70599 Stuttgart, Germany
✉ Hans Peter Maurer, email: h-p.maurer@uni-hohenheim.de

Supplementary material

Supplementary Table S1 Coordinates, altitude and experimental design for the four locations.

Location	Coordinates	Altitude (m)	Design	
			2018	2019
Hohenheim	48°42'53''N, 9°11'16''E	400	row-column	row-column
Ihinger Hof	48°44'40''N, 8°55'25''E	480	incomplete blocks	row-column
Eckartsweier	48°31'17''N, 7°52'13''E	140	incomplete blocks	row-column
Moosburg	48°26'36''N, 11°54'22''E	420	row-column	row-column

Supplementary Table S2 Preceding crop, plant-available nitrogen, soil mineral nitrogen and yield potential based on the previous three years at the four locations in 2018 and 2019.

Environment		Preceding crop	Plant available nitrogen				Soil mineral nitrogen					Yield potential
							spring	after harvest				
			N1	N2	N3	N4		N1	N2	N3	N4	
			[kg(N) ha^{-1}]									[dt ha^{-1}]
Hohenheim	2018	soybean	88	154	220	286	30	32	25	26	31	102
Ihinger Hof	2018	cereals	80	140	200	260	21	18	9	13	37	84
Eckartsweier	2018	maize	80	140	200	260	30	39	40	37	44	85
Moosburg	2018	cereals	88	154	220	286	22	63	53	67	74	108
Hohenheim	2019	winter rape	88	154	220	286	70	12	16	13	19	102
Ihinger Hof	2019	sugar beet	80	140	200	260	24	10	11	18	20	87
Eckartsweier	2019	maize	80	140	200	260	84	16	16	29	28	87
Moosburg	2019	maize	88	154	220	286	44	59	67	59	61	100

Supplementary Table S3 Overall BLUES for grain yield and protein content of 126 registered cultivars included in this study. This table is provided as separate Excel file.

Supplementary Table S4 Summary of the results from genome-wide association mapping.

QTL	diagnostic Marker	Chr.	Pos. (physical)	*P* value	p_G joint‡	p_G single§	Effect	p†
N1								
Grain yield (45.13 % p_G total)								
q1B.B	S10520644¶	1B	33073336	7.62×10^{-7}	0.71	12.56	-7.71	0.93
q1B.C	D3622528¶	1B	43957149	6.01×10^{-7}	0.64	18.10	8.07	0.88
q1B.A	S8537769¶	1B	675825821	7.74×10^{-9}	10.90	10.90	8.98	0.94
q4A.A	D8514460	4A	361463133	5.14×10^{-7}	1.04	11.79	5.39	0.81
q5A.A	S15998366	5A	548507670	6.77×10^{-8}	8.26	11.54	8.96	0.94
q5A.B	D54349290¶	5A	569174428	7.55×10^{-8}	12.11	18.76	-8.42	0.88
q5B.A	D54358656	5B	473192647	2.89×10^{-7}	1.38	10.84	6.90	0.90
q5B.C	D54359459¶	5B	544925204	4.53×10^{-7}	0.02	11.43	-5.89	0.85
q6B.A	D8523323	6B	125484746	5.18×10^{-7}	0.99	19.98	-9.28	0.90
q6R.A	D8517619	6R	827791678	3.10×10^{-8}	11.28	8.79	5.34	0.86
q7B.A	D4550121	7B	701924179	6.23×10^{-8}	0.05	7.38	4.71	0.85
Starch content (12.07 % p_G total)								
q4A.B	D10500730	4A	616120256	2.02×10^{-7}	5.13	5.13	-0.74	0.91
	D8531741	4A	681683099	8.99×10^{-7}	3.48	3.83	-0.39	0.35
q5B.D	S4208113	5B	455738742	5.86×10^{-7}	4.33	6.4	-0.63	0.84
Protein yield (10.23 % p_G total)								
q1B.A	S8537769¶	1B	675825821	2.89×10^{-7}	10.84	10.84	0.57	0.94
Nitrogen use efficiency (42.45 % p_G total)								
q1B.B	S10520644	1B	33073336	1.07×10^{-7}	6.35	15.19	-0.09	0.93
q1B.A	S8537769¶	1B	675825821	8.47×10^{-7}	1.72	8.33	0.08	0.94
q4A.A	D8514460	4A	361463133	4.92×10^{-8}	15.81	11.82	0.06	0.81
q5A.B	D54349290¶	5A	569174428	5.88×10^{-7}	7.49	17.90	-0.09	0.88
q6R.A	D8517619	6R	827791678	4.16×10^{-10}	10.70	10.70	0.06	0.86
q7B.A	D14479486¶	7B	701924179	1.87×10^{-7}	0.56	9.41	0.06	0.84
Nitrogen use efficiency$_{PC}$ (20.81 % p_G total)								
q1B.B	S10520644¶	1B	33073336	6.90×10^{-7}	16.49	16.49	7.31×10^{-3}	0.07
q2A.A	D10515912	2A	3779241	7.05×10^{-7}	4.56	16.42	5.21×10^{-3}	0.18
q2B.A	D4214705	2B	5680494	9.41×10^{-7}	0.59	15.79	5.11×10^{-3}	0.18
N2								
Grain yield (59.57% p_G total)								
q1B.B	S10520644¶	1B	33073336	3.81×10^{-7}	0.57	15.12	-10.45	0.93
q1B.C	D4343597¶	1B	43957149	9.72×10^{-7}	0.17	15.08	-6.91	0.75
q1B.A	S8537769¶	1B	675825821	2.52×10^{-8}	3.17	13.21	12.01	0.94
q2B.B	D3047892	2B	3907859	1.66×10^{-7}	2.27	8.23	8.66	0.93
q4B.A	D8516040¶	4B	11349918	2.77×10^{-8}	2.20	25.73	-12.81	0.90
q5A.A	S15998366	5A	548507670	4.94×10^{-9}	6.98	15.04	12.74	0.94
q5A.B	D54349290¶	5A	569174428	2.31×10^{-9}	2.11	24.02	-11.83	0.88
q5B.A	D54358656	5B	473192647	2.67×10^{-8}	1.13	14.82	10.02	0.90
q5B.B	D14468323¶	5B	535028261	8.76×10^{-10}	4.66	26.49	-12.53	0.89
q5B.C	D3615140	5B	563543624	5.21×10^{-11}	21.99	21.99	-11.73	0.89
q6B.A	D8523323	6B	125484746	2.38×10^{-8}	1.87	25.31	-12.96	0.90
q6R.A	D8517619	6R	827791678	6.83×10^{-8}	13.26	7.31	6.05	0.86
q7A.A	D54356766	7A	250632562	2.62×10^{-8}	0.01	25.45	-12.51	0.89
q7B.A	D4550121	7B	701924179	2.87×10^{-7}	0.02	5.25	4.93	0.85

Protein content (39.01% p_G total)								
q1A.A	D54353737¶	1A	22189802	3.88×10^{-7}	0.06	15.64	0.72	0.07
q1A.B	D10514043	1A	439067205	1.13×10^{-7}	0.53	7.89	0.34	0.17
q1B.B	S10520644¶	1B	33073336	1.04×10^{-9}	22.90	22.90	0.80	0.07
q1B.C	D3622528¶	1B	43957149	4.34×10^{-7}	0.08	19.86	-0.62	0.12
q2B.A	D10501541¶	2B	5699265	6.33×10^{-7}	1.14	14.96	0.49	0.15
q3A.A	D3615571¶	3A	94113507	6.98×10^{-7}	0.01	6.14	0.30	0.17
q4A.A	D8514460	4A	361463133	1.52×10^{-8}	0.44	14.28	-0.44	0.19
q4A.E	D4547587	4A	621483588	3.01×10^{-7}	1.18	14.80	-0.66	0.08
q4B.A	D3624064¶	4B	11349918	1.43×10^{-7}	0.71	25.00	0.76	0.10
q5A.A	S15998366	5A	548507670	6.93×10^{-7}	3.08	14.88	-0.75	0.06
q5B.A	D54358656	5B	473192647	5.30×10^{-7}	0.19	10.96	-0.51	0.10
q5B.B	D14468323¶	5B	535028261	4.75×10^{-8}	1.62	23.51	0.70	0.11
q5B.C	D11912849	5B	563463729	1.18×10^{-8}	8.10	20.83	0.68	0.11
q6B.A	D8523323	6B	125484746	5.62×10^{-8}	0.34	21.46	0.71	0.10
Starch content (18.74% p_G total)								
	D4552423¶	1A	535464070	4.48×10^{-7}	6.99	10.97	0.82	0.81
q4A.B	D10500730	4A	616120256	2.75×10^{-8}	6.48	6.48	-0.85	0.91
q5B.D	S4208113	5B	455738742	2.06×10^{-7}	5.20	7.81	-0.70	0.84
Protein yield (18.53% p_G total)								
q5A.B	D3605414¶	5A	562633844	4.45×10^{-7}	4.69	17.36	-0.80	0.90
q5B.C	D3615140	5B	563543624	2.25×10^{-7}	14.40	14.40	-0.70	0.89
Nitrogen use efficiency (51.33% p_G total)								
q1B.B	S10520644¶	1B	33073336	6.30×10^{-8}	5.60	18.19	-0.07	0.93
q1B.C	D54353627¶	1B	43957149	8.81×10^{-7}	0.01	23.27	-0.08	0.90
q4A.A	D8514460	4A	361463133	4.06×10^{-7}	0.33	13.30	0.04	0.81
q4B.A	D8516040¶	4B	11349918	1.94×10^{-7}	0.85	24.88	-0.08	0.90
q5A.B	D54349290¶	5A	569174428	1.16×10^{-7}	1.77	22.65	-0.07	0.88
q5B.A	D54358656	5B	473192647	6.06×10^{-8}	2.98	13.80	0.06	0.90
q5B.B	D14468323¶	5B	535028261	8.84×10^{-9}	6.52	26.08	-0.08	0.89
q5B.C	D3615140	5B	563543624	1.89×10^{-9}	23.54	20.74	-0.07	0.89
q6B.A	D8523323	6B	125484746	3.53×10^{-7}	0.33	24.48	-0.08	0.90
q6R.A	D54345506¶	6R	828526410	5.34×10^{-10}	9.89	9.89	0.04	0.85
q7A.A	D54356766	7A	250632562	2.28×10^{-7}	0.31	24.76	-0.08	0.89
q7B.A	D14479486¶	7B	701924179	1.03×10^{-6}	0.35	7.39	0.04	0.84
Nitrogen use efficiency$_{PC}$ (38.43% p_G total)								
q1A.A	D54353737¶	1A	22189802	3.32×10^{-7}	0.09	15.57	4.93×10^{-3}	0.07
q1A.B	D10514043	1A	439067205	1.02×10^{-7}	0.35	7.67	2.31×10^{-3}	0.17
q1B.B	S10520644¶	1B	33073336	8.11×10^{-10}	22.58	22.58	5.50×10^{-3}	0.07
q1B.C	D3622528¶	1B	43957149	1.03×10^{-6}	0.22	19.40	-4.22×10^{-3}	0.12
q2B.A	D10501541¶	2B	5699265	6.71×10^{-7}	1.16	14.97	3.39×10^{-3}	0.15
q3A.A	D3615571¶	3A	94113507	6.13×10^{-7}	0.06	5.96	2.03×10^{-3}	0.17
q4A.A	D8514460	4A	361463133	3.96×10^{-8}	0.35	13.67	-2.93×10^{-3}	0.19
q4A.E	D4547587	4A	621483588	3.25×10^{-7}	1.52	15.12	-4.58×10^{-3}	0.08
q4B.A	D3624064¶	4B	11349918	2.29×10^{-7}	0.74	24.70	5.16×10^{-3}	0.10
q5A.A	S15998366	5A	548507670	8.44×10^{-7}	2.80	14.63	-5.11×10^{-3}	0.06
q5B.A	D54358656	5B	473192647	5.31×10^{-7}	0.26	11.07	-3.53×10^{-3}	0.10
q5B.B	D14468323¶	5B	535028261	8.14×10^{-8}	1.71	23.26	4.78×10^{-3}	0.11
q5B.C	D11912849	5B	563463729	2.29×10^{-8}	7.94	20.46	4.61×10^{-3}	0.11
q6B.A	D8523323	6B	125484746	1.13×10^{-7}	0.49	21.16	4.83×10^{-3}	0.10

N3								
Grain yield (50.95 % p_G total)								
q1B.B	S10520644¶	1B	33073336	1.50×10^{-8}	4.26	16.23	-12.11	0.93
q1B.C	D54353627¶	1B	43957149	4.88×10^{-7}	0.10	21.41	-12.69	0.90
q1B.A	S8537769¶	1B	675825821	1.67×10^{-8}	3.00	12.32	12.54	0.94
q2B.B	D3047892	2B	3907859	6.89×10^{-7}	0.96	6.41	8.31	0.93
q4A.A	D8514460	4A	361463133	2.59×10^{-7}	0.41	14.77	8.13	0.81
q4B.A	D3624064¶	4B	11349918	9.47×10^{-7}	0.44	22.60	-13.17	0.90
q5A.A	S15998366	5A	548507670	2.72×10^{-8}	2.54	13.39	13.12	0.94
q5A.B	D54349290¶	5A	569174428	2.53×10^{-7}	1.22	19.81	-11.67	0.88
q5B.A	D54358656	5B	473192647	1.41×10^{-8}	2.07	12.88	10.15	0.90
q5B.B	D14468323¶	5B	535028261	7.79×10^{-9}	23.05	23.05	-12.70	0.89
q5B.C	D11912849	5B	563463729	1.32×10^{-7}	1.77	19.31	-11.94	0.89
q6B.A	D8523323	6B	125484746	9.07×10^{-8}	0.51	22.67	-13.32	0.90
q6R.A	D54345506¶	6R	828526410	1.82×10^{-7}	12.09	7.83	6.58	0.85
Protein content (47.92 % p_G total)								
q1A.A	D54353737¶	1A	22189802	1.27×10^{-7}	1.80	15.78	0.79	0.07
	D54353116¶	1A	65987966	8.63×10^{-7}	0.27	10.10	0.47	0.13
q1A.C	D54353523	1A	321114051	8.12×10^{-7}	0.12	19.16	0.78	0.09
q1A.B	S10519795¶	1A	482783227	9.13×10^{-7}	0.46	9.90	0.35	0.29
q1B.B	S10520644¶	1B	33073336	6.78×10^{-11}	23.22	23.22	0.88	0.07
q1B.C	D3622528¶	1B	43957149	5.55×10^{-9}	0.88	20.85	-0.70	0.12
q1B.D	D36891642¶	1B	561500206	2.58×10^{-7}	0.12	17.51	-0.63	0.13
q2A.A	D10515912	2A	3779241	2.63×10^{-7}	0.50	20.98	0.60	0.18
q2B.A	D4214705	2B	5680494	8.15×10^{-7}	0.24	18.86	0.57	0.18
q3A.B	D11911475	3A	655894084	2.53×10^{-7}	7.94	19.50	0.45	0.57
q4A.A	D8514460	4A	361463133	1.61×10^{-8}	0.17	14.24	-0.48	0.19
q4B.A	D3624064¶	4B	11349918	4.75×10^{-8}	0.42	24.71	0.82	0.10
q4B.B	D54357858¶	4B	106030667	7.59×10^{-7}	0.19	19.68	0.80	0.08
q5A.A	S15998366	5A	548507670	3.07×10^{-8}	3.46	14.66	-0.82	0.06
q5A.B	D3624780¶	5A	569066839	2.46×10^{-7}	0.28	20.48	0.69	0.12
q5B.D	S4208113	5B	455738742	6.02×10^{-7}	0.90	14.41	0.47	0.16
q5B.A	D54358656	5B	473192647	2.37×10^{-7}	0.31	11.28	-0.57	0.10
q5B.B	D14468323¶	5B	535028261	7.98×10^{-10}	9.86	23.75	0.77	0.11
q5B.C	D3615140	5B	563543624	7.33×10^{-9}	0.74	20.82	0.74	0.11
q6B.A	D8523323	6B	125484746	5.77×10^{-7}	0.26	20.46	0.76	0.10
Starch content (17.21 % p_G total)								
q3A.B	D11911475	3A	655894084	4.48×10^{-7}	4.20	7.46	-0.55	0.43
q5A.C	S11910144	5A	493025678	1.97×10^{-8}	13.53	13.53	-0.98	0.82
q5A.D	D4218466¶	5A	512126150	3.55×10^{-7}	0.09	8.09	0.69	0.79
q5B.D	D4220478¶	5B	454491307	8.63×10^{-7}	1.15	10.08	-0.83	0.83
Nitrogen use efficiency (51.83 % p_G total)								
q1B.B	S10520644¶	1B	33073336	1.81×10^{-8}	4.15	16.21	-0.06	0.93
q1B.C	D54353627¶	1B	43957149	4.16×10^{-7}	0.11	21.85	-0.06	0.90
q1B.A	S8537769¶	1B	675825821	2.40×10^{-8}	2.94	12.28	0.06	0.94
q2B.B	D3047892	2B	3907859	3.93×10^{-7}	1.05	6.73	0.04	0.93
q4A.A	D8514460	4A	361463133	1.54×10^{-7}	0.38	15.21	0.04	0.81
q4A.C	D10496248	4A	518980831	6.99×10^{-7}	0.01	9.12	0.03	0.87
q4B.A	D3624064¶	4B	11349918	7.84×10^{-7}	0.40	23.00	-0.06	0.90
q5A.A	S15998366	5A	548507670	5.08×10^{-8}	2.51	13.36	0.06	0.94
q5A.B	D54349290¶	5A	569174428	2.62×10^{-7}	0.93	19.96	-0.05	0.88

q5B.A	D54358656	5B	473192647	4.09×10^{-9}	13.50	13.50	0.05	0.90
q5B.B	D14468323[¶]	5B	535028261	5.03×10^{-9}	12.27	23.48	-0.06	0.89
q5B.C	D11912849	5B	563463729	7.66×10^{-8}	1.76	19.74	-0.06	0.89
q6B.A	D8523323	6B	125484746	5.81×10^{-8}	0.53	23.15	-0.06	0.90
q6R.A	D54345506[¶]	6R	828526410	2.04×10^{-7}	12.44	7.84	0.03	0.85
Nitrogen use efficiency$_{PC}$ (45.79 % p_G total)								
q1A.A	D54353737[¶]	1A	22189802	1.21×10^{-7}	1.63	15.97	3.85×10^{-3}	0.07
q1A.C	D54353523	1A	321114051	2.41×10^{-7}	0.34	19.40	3.80×10^{-3}	0.09
q1A.B	D10514043	1A	439067205	5.90×10^{-7}	0.19	7.12	1.71×10^{-3}	0.17
q1B.B	S10520644[¶]	1B	33073336	5.62×10^{-11}	23.18	23.18	4.25×10^{-3}	0.07
q1B.C	D3622528[¶]	1B	43957149	5.46×10^{-8}	0.25	19.35	-3.25×10^{-3}	0.12
q1B.D	D36891642[¶]	1B	561500206	4.22×10^{-7}	0.09	17.48	-3.04×10^{-3}	0.13
q2A.A	D10515912	2A	3779241	4.78×10^{-7}	0.42	20.62	2.88×10^{-3}	0.18
q3A.B	D11911475	3A	655894084	3.56×10^{-7}	7.57	19.16	2.14×10^{-3}	0.57
q4A.A	D8514460	4A	361463133	1.54×10^{-8}	0.36	14.13	-2.30×10^{-3}	0.19
q4B.A	D3624064[¶]	4B	11349918	1.19×10^{-7}	0.56	23.81	3.91×10^{-3}	0.10
q4B.B	D54357858[¶]	4B	106030667	4.26×10^{-7}	0.11	19.79	3.89×10^{-3}	0.08
q5A.A	S15998366	5A	548507670	3.38×10^{-8}	2.86	14.60	-3.95×10^{-3}	0.06
q5B.D	D4208540[¶]	5B	448129453	5.81×10^{-7}	0.35	16.92	2.68×10^{-3}	0.17
q5B.A	D54358656	5B	473192647	2.14×10^{-7}	0.30	11.05	-2.71×10^{-3}	0.10
q5B.B	D14468323[¶]	5B	535028261	2.58×10^{-9}	9.23	22.84	3.65×10^{-3}	0.11
q5B.C	D3615140	5B	563543624	5.14×10^{-8}	1.52	19.66	3.48×10^{-3}	0.11
N4								
Grain yield (38.71 % p_G total)								
q1B.B	S10520644[¶]	1B	33073336	4.76×10^{-7}	2.00	14.93	-11.55	0.93
q1B.A	S8537769[¶]	1B	675825821	3.06×10^{-7}	4.28	11.28	12.58	0.94
q4A.C	D10496248	4A	518980831	1.00×10^{-7}	3.09	10.27	8.19	0.87
q5B.B	D14468323[¶]	5B	535028261	8.04×10^{-8}	20.52	20.52	-12.44	0.89
q5B.C	D11912849	5B	563463729	1.09×10^{-7}	0.05	17.30	-11.73	0.89
q6R.A	D54345506[¶]	6R	828526410	1.36×10^{-7}	10.32	7.19	6.54	0.85
Protein content (53.32 % p_G total)								
q1A.A	D54353631	1A	22332562	5.97×10^{-7}	0.91	18.75	0.79	0.08
q1A.C	D54353523	1A	321114051	6.44×10^{-8}	1.14	17.05	0.72	0.09
q1B.B	S10520644[¶]	1B	33073336	4.47×10^{-10}	19.73	19.73	0.78	0.07
q1B.C	D3622528[¶]	1B	43957149	1.05×10^{-8}	0.50	18.32	-0.64	0.12
q1B.D	D36891642[¶]	1B	561500206	2.12×10^{-7}	0.41	14.50	-0.56	0.13
q3A.B	D11911475	3A	655894084	2.71×10^{-7}	5.84	22.15	0.47	0.57
q4A.A	D8514460	4A	361463133	1.97×10^{-7}	<0.01	11.04	-0.41	0.19
q4A.D	D4202540	4A	583019227	2.81×10^{-7}	0.02	15.20	-0.50	0.18
q4B.A	D3624064[¶]	4B	11349918	1.42×10^{-8}	0.75	22.69	0.77	0.10
q5A.A	S15998366	5A	548507670	3.33×10^{-7}	1.52	13.15	-0.72	0.06
q5A.B	D3624780[¶]	5A	569066839	5.38×10^{-7}	0.39	17.43	0.63	0.12
q5B.D	D36886216	5B	447844712	5.54×10^{-7}	0.39	23.34	0.50	0.35
q5B.B	D14468323[¶]	5B	535028261	1.60×10^{-9}	1.71	20.93	0.71	0.11
q5B.C	D11912849	5B	563463729	4.54×10^{-10}	7.23	18.29	0.68	0.11
q5R.A	D4215260	5R	593836518	1.41×10^{-7}	5.02	22.62	0.49	0.36
q6B.A	D8523323	6B	125484746	3.82×10^{-8}	0.33	19.52	0.72	0.10
q6B.B	D4204808[¶]	6B	611280050	5.28×10^{-8}	7.22	13.54	0.45	0.79
Starch content (18.44 % p_G total)								
q3A.B	D11911475	3A	655894084	1.59×10^{-7}	6.12	9.63	-0.58	0.43
q5A.C	S11910144	5A	493025678	1.12×10^{-7}	4.30	12.07	-0.87	0.82

q5A.D	D4218466[¶]	5A	512126150	2.48×10^{-7}	0.24	7.40	0.62	0.79
q5B.D	S4208113	5B	455738742	3.86×10^{-8}	8.45	8.45	-0.68	0.84
Nitrogen use efficiency (39.29 % p_G total)								
q1B.B	S10520644[¶]	1B	33073336	4.89×10^{-7}	1.93	14.88	-0.04	0.93
q1B.A	S8537769[¶]	1B	675825821	1.38×10^{-7}	6.70	11.62	0.05	0.94
q4A.C	D10496248	4A	518980831	1.39×10^{-7}	1.78	10.38	0.03	0.87
q5B.B	D14468323[¶]	5B	535028261	1.15×10^{-7}	22.62	20.13	-0.04	0.89
q5B.C	D11912849	5B	563463729	1.39×10^{-7}	0.06	16.88	-0.04	0.89
q6R.A	D54345506[¶]	6R	828526410	8.89×10^{-8}	7.77	7.77	0.02	0.85
Nitrogen use efficiency$_{PC}$ (52.10 % p_G total)								
q1A.A	D54353737[¶]	1A	22189802	3.95×10^{-7}	0.88	13.28	2.63×10^{-3}	0.07
q1A.C	D54353523	1A	321114051	3.60×10^{-8}	0.81	17.21	2.69×10^{-3}	0.09
q1B.B	S10520644[¶]	1B	33073336	2.91×10^{-10}	19.93	19.93	2.91×10^{-3}	0.07
q1B.C	D3622528[¶]	1B	43957149	9.81×10^{-9}	0.71	18.48	-2.39×10^{-3}	0.12
q1B.D	D36891642[¶]	1B	561500206	9.93×10^{-8}	0.28	14.14	-2.05×10^{-3}	0.13
q3A.B	D11911475	3A	655894084	4.67×10^{-7}	4.98	21.84	1.72×10^{-3}	0.57
q4A.A	D8514460	4A	361463133	1.27×10^{-7}	0.02	11.06	-1.53×10^{-3}	0.19
q4A.D	D4202540	4A	583019227	1.74×10^{-7}	0.02	15.53	-1.87×10^{-3}	0.18
q4B.A	D3613220[¶]	4B	11349918	7.55×10^{-7}	0.83	19.09	2.60×10^{-3}	0.10
q5A.B	D3624780[¶]	5A	569066839	8.82×10^{-7}	0.71	17.36	2.31×10^{-3}	0.12
q5B.D	D36886216	5B	447844712	2.14×10^{-7}	0.41	24.59	1.89×10^{-3}	0.35
q5B.B	D14468323[¶]	5B	535028261	3.47×10^{-9}	1.68	20.44	2.59×10^{-3}	0.11
q5B.C	D11912849	5B	563463729	1.01×10^{-9}	6.85	17.81	2.49×10^{-3}	0.11
q5R.A	D4215260	5R	593836518	4.18×10^{-8}	5.72	23.84	1.85×10^{-3}	0.36
q6B.A	D8523323	6B	125484746	5.86×10^{-8}	0.55	19.07	2.65×10^{-3}	0.10
q6B.B	D4204808[¶]	6B	611280050	3.67×10^{-8}	7.65	13.50	1.65×10^{-3}	0.79
Overall								
Grain yield (53.04 % p_G total)								
q1B.B	S10520644[¶]	1B	33073336	3.74×10^{-8}	1.43	15.38	-10.62	0.93
q1B.C	D3622528[¶]	1B	43957149	7.66×10^{-7}	<0.01	19.02	10.29	0.88
q1B.A	S8537769[¶]	1B	675825821	4.83×10^{-9}	12.21	12.21	11.66	0.94
q2B.B	D3047892	2B	3907859	7.71×10^{-8}	1.93	7.14	8.09	0.93
q4A.A	D8514460	4A	361463133	1.05×10^{-7}	0.24	13.92	7.28	0.81
q4A.C	D10496248	4A	518980831	1.93×10^{-7}	0.02	9.18	6.89	0.87
q4A.E	D4547587	4A	621483588	9.33×10^{-7}	0.39	13.18	10.53	0.92
q4B.A	D54360444	4B	11349918	9.25×10^{-7}	1.14	20.41	-11.55	0.90
q5A.A	S15998366	5A	548507670	1.07×10^{-8}	3.68	13.05	11.89	0.94
q5A.B	D54349290[¶]	5A	569174428	9.18×10^{-8}	1.04	19.84	-10.77	0.88
q5B.A	D54358656	5B	473192647	4.83×10^{-8}	1.48	11.99	9.03	0.90
q5B.B	D14468323[¶]	5B	535028261	6.19×10^{-9}	3.66	22.57	-11.59	0.89
q5B.C	D3615140	5B	563543624	5.21×10^{-9}	12.18	18.58	-10.81	0.89
q6B.A	D8523323	6B	125484746	5.94×10^{-8}	1.01	22.29	-12.19	0.90
q6R.A	D54345506[¶]	6R	828526410	1.65×10^{-8}	13.40	9.02	6.51	0.85
q7B.A	D4550121	7B	701924179	8.76×10^{-8}	<0.01	6.15	5.35	0.85
Protein content (45.16 % p_G total)								
q1A.A	D54353737[¶]	1A	22189802	1.57×10^{-7}	0.49	14.61	0.71	0.07
q1A.C	D54353523	1A	321114051	9.00×10^{-7}	0.12	18.12	0.71	0.09
q1A.B	S10519795[¶]	1A	482783227	3.23×10^{-7}	0.71	8.68	0.31	0.29
q1B.B	S10520644[¶]	1B	33073336	3.94×10^{-10}	21.34	21.34	0.79	0.07
q1B.C	D3622528[¶]	1B	43957149	1.38×10^{-8}	0.43	19.59	-0.63	0.12
q1B.D	D36891642[¶]	1B	561500206	2.20×10^{-7}	0.06	16.29	-0.57	0.13

q2A.A	D10515912	2A	3779241	2.31×10^{-7}	0.31	19.19	0.54	0.18
q2B.A	D4214705	2B	5680494	5.27×10^{-7}	0.15	17.68	0.52	0.18
q3A.B	D11911475	3A	655894084	8.52×10^{-7}	7.72	18.32	0.41	0.57
q4A.A	D8514460	4A	361463133	9.17×10^{-9}	0.35	13.57	-0.44	0.19
q4A.D	D4202540	4A	583019227	8.18×10^{-7}	<0.01	17.86	-0.52	0.18
q4B.A	D3624064¶	4B	11349918	4.98×10^{-8}	0.58	23.81	0.76	0.10
q5A.A	S15998366	5A	548507670	3.43×10^{-8}	3.12	14.01	-0.74	0.06
q5A.B	D3624780¶	5A	569066839	5.59×10^{-7}	0.08	19.29	0.63	0.12
q5B.A	D54358656	5B	473192647	1.98×10^{-7}	0.09	9.99	-0.50	0.10
q5B.D	D36891806	5B	448891481	3.90×10^{-7}	1.34	14.42	0.48	0.16
q5B.B	D14468323¶	5B	535028261	2.95×10^{-9}	1.24	22.37	0.70	0.11
q5B.C	D3615140	5B	563543624	2.85×10^{-9}	8.57	20.07	0.68	0.11
q6B.A	D8523323	6B	125484746	6.71×10^{-8}	0.30	20.22	0.71	0.10
Starch content (13.28 % p_G total)								
q4A.B	D10500730	4A	616120256	8.31×10^{-8}	3.19	5.66	-0.78	0.91
q5A.C	S11910144	5A	493025678	1.75×10^{-7}	2.89	12.02	-0.89	0.82
q5B.D	S4208113	5B	455738742	4.36×10^{-8}	8.07	8.07	-0.71	0.84
Protein yield (17.63 % p_G total)								
q1B.A	S8537769¶	1B	675825821	7.28×10^{-7}	12.24	10.15	0.83	0.94
q6R.A	D54345506¶	6R	828526410	3.07×10^{-7}	6.13	6.13	0.41	0.85
Nitrogen use efficiency (55.34 % p_G total)								
q1B.B	S10520644¶	1B	33073336	1.19×10^{-8}	5.63	16.49	-0.06	0.93
q1B.C	D54353627¶	1B	43957149	7.31×10^{-7}	0.18	21.68	-0.06	0.90
q1B.A	S8537769¶	1B	675825821	3.07×10^{-8}	3.58	11.74	0.06	0.94
q2B.B	D3047892	2B	3907859	2.48×10^{-7}	1.30	6.79	0.04	0.93
q4A.A	D8514460	4A	361463133	5.27×10^{-8}	0.82	14.86	0.04	0.81
q4A.C	D10496248	4A	518980831	7.59×10^{-8}	0.05	9.61	0.04	0.87
q4B.A	D8516040¶	4B	11349918	9.97×10^{-7}	0.84	22.70	-0.06	0.90
q5A.A	S15998366	5A	548507670	3.76×10^{-8}	3.04	12.83	0.06	0.94
q5A.B	D54349290¶	5A	569174428	2.70×10^{-7}	0.51	19.98	-0.06	0.88
q5B.A	D54358656	5B	473192647	2.66×10^{-8}	1.81	12.35	0.05	0.90
q5B.B	D14468323¶	5B	535028261	2.56×10^{-9}	26.65	23.64	-0.06	0.89
q5B.C	D54359459¶	5B	544925204	1.94×10^{-7}	0.30	14.64	-0.04	0.85
q6B.A	D8523323	6B	125484746	4.62×10^{-8}	1.01	23.02	-0.06	0.90
q6R.A	D54345506¶	6R	828526410	1.47×10^{-9}	9.62	9.62	0.04	0.85
q7B.A	D14479486¶	7B	701924179	7.67×10^{-7}	0.74	7.15	0.03	0.84
Nitrogen use efficiency$_{PC}$ (46.45 % p_G total)								
q1A.A	D54353737¶	1A	22189802	8.42×10^{-8}	0.40	15.65	4.02×10^{-3}	0.07
q1A.C	D54353523	1A	321114051	1.94×10^{-7}	0.67	19.39	4.02×10^{-3}	0.09
q1A.B	D10514043	1A	439067205	1.38×10^{-7}	0.40	6.67	1.75×10^{-3}	0.17
q1B.B	S10520644¶	1B	33073336	2.25×10^{-10}	22.56	22.56	4.44×10^{-3}	0.07
q1B.C	D3622528¶	1B	43957149	3.42×10^{-8}	0.46	19.98	-3.49×10^{-3}	0.12
q1B.D	D36891642¶	1B	561500206	1.04×10^{-7}	0.09	16.42	-3.12×10^{-3}	0.13
q2A.A	D10515912	2A	3779241	3.80×10^{-7}	0.36	19.68	2.98×10^{-3}	0.18
q2B.A	D4214705	2B	5680494	6.83×10^{-7}	0.11	17.91	2.84×10^{-3}	0.18
q3A.A	D3615571¶	3A	94113507	9.41×10^{-7}	0.08	5.22	1.55×10^{-3}	0.17
q3A.B	D11911475	3A	655894084	6.96×10^{-7}	7.04	19.01	2.26×10^{-3}	0.57
q4A.A	D8514460	4A	361463133	8.12×10^{-9}	0.31	13.80	-2.40×10^{-3}	0.19
q4B.A	D3624064¶	4B	11349918	5.03×10^{-8}	0.59	24.27	4.17×10^{-3}	0.10
q5A.A	S15998366	5A	548507670	4.84×10^{-8}	3.14	14.34	-4.07×10^{-3}	0.06
q5A.B	D54349290¶	5A	569174428	5.30×10^{-7}	0.07	17.89	3.39×10^{-3}	0.12

q5B.D	S4208113	5B	455738742	9.76×10^{-7}	1.30	12.73	2.32×10^{-3}	0.16
q5B.A	D54358656	5B	473192647	1.22×10^{-7}	0.05	10.15	-2.75×10^{-3}	0.10
q5B.B	D14468323[¶]	5B	535028261	2.53×10^{-9}	9.43	22.87	3.86×10^{-3}	0.11
q5B.C	D3615140	5B	563543624	6.62×10^{-9}	0.95	20.30	3.74×10^{-3}	0.11
q6B.A	D8523323	6B	125484746	8.55×10^{-8}	0.30	20.61	3.88×10^{-3}	0.10

[†]Frequency of the allele increasing the trait value

[‡] p_G values were obtained by a joint fit of all significant makers for the respective trait in a linear model

[§] p_G values were obtained by fitting each single significant marker for the respective trait in a linear model

[¶] Unmapped marker that was placed on the map based on its LD with mapped markers

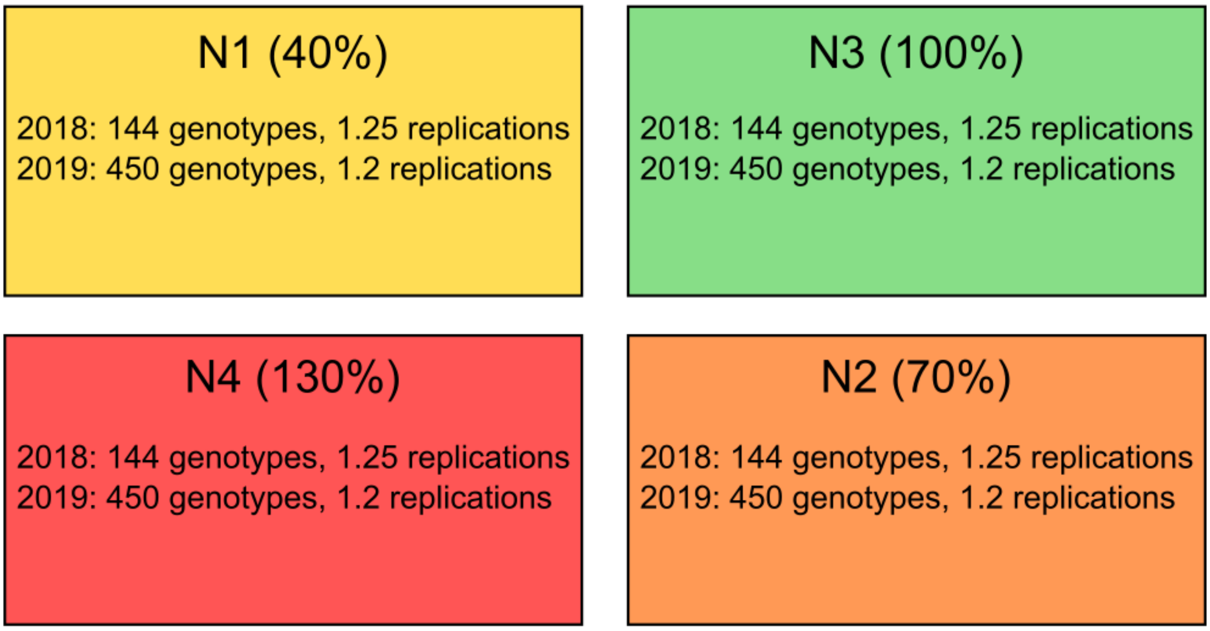

Supplementary Fig. S1 Exemplary representation of the field design used at the single environments in 2018 and 2019.

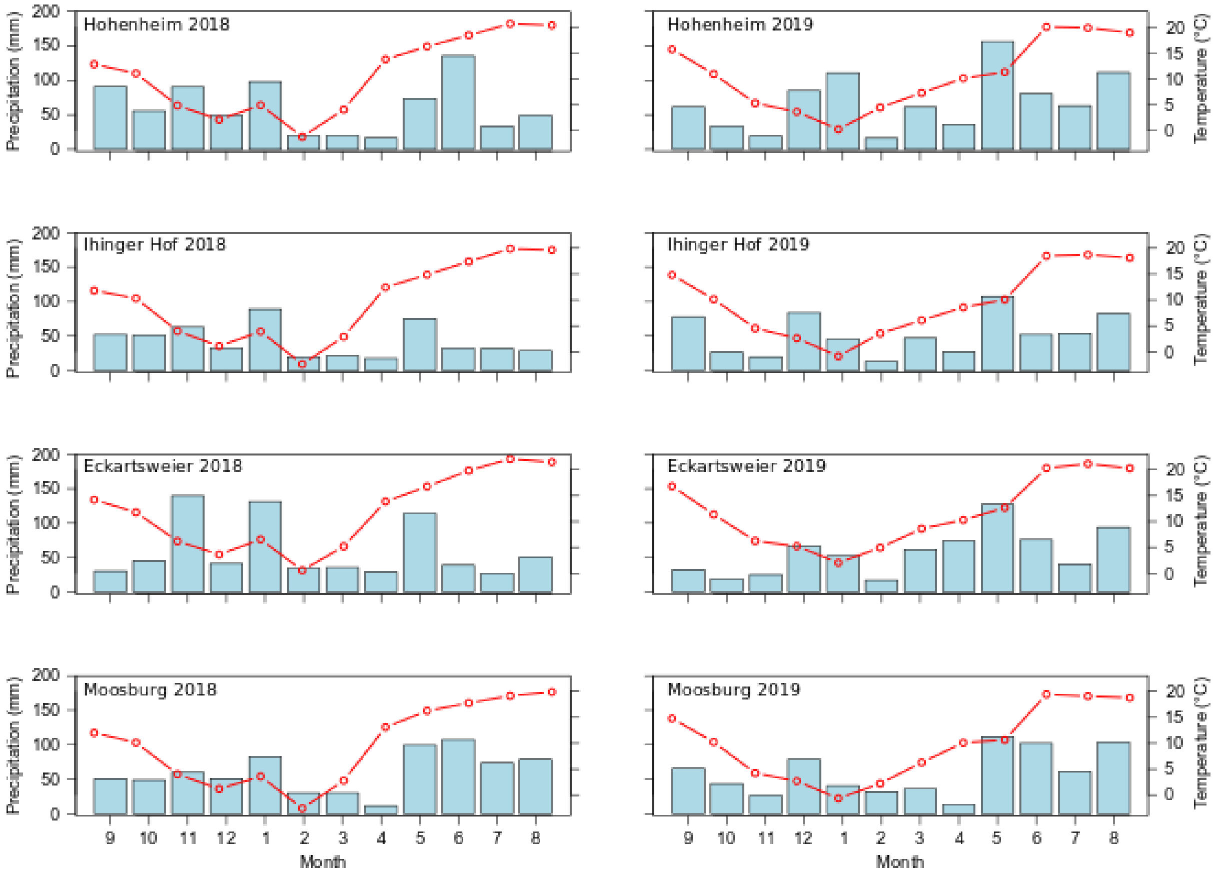

Supplementary Fig. S2 Monthly precipitation (mm) and mean temperature (°C) during the growing season 2018 and 2019 at the different locations. Data was provided by the Center for Agricultural Technology Augustenberg for environments Hohenheim, Ihinger Hof and Eckartsweier in 2018 and 2019 as well as by the German Weather Service (DWD) for environments Moosburg nearby weather station in 2018 and 2019.

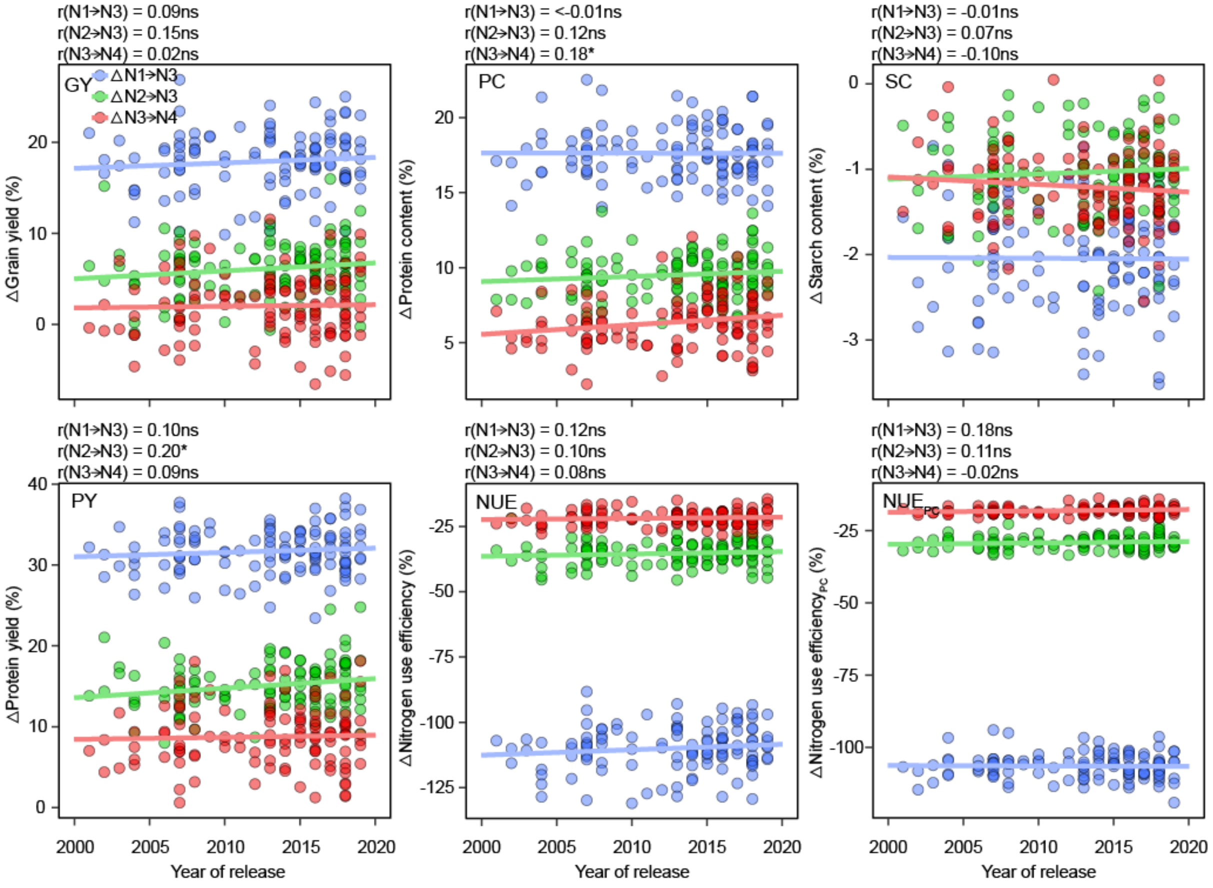

Supplementary Fig. S3 Temporal trends of the relative effect of additional fertilizer on the performance of 126 triticale cultivars, registered over the last 20 years. The legal maximum for conventional farmers in Germany (nitrogen fertilization level N3) is used as a reference.
